# Tumor-Responsive Delivery of 5‑Fluorouracil from a Redox-Triggered Supramolecular Gelator

**DOI:** 10.1021/acs.bioconjchem.6c00108

**Published:** 2026-08-10

**Authors:** Marko Mihajlovic, Milos Mihajlovic, Paola Alletto, Slavko Kralj, Natalia Rosso, Silvia Marchesan

**Affiliations:** † Department of Chemical and Pharmaceutical Sciences, 9315 University of Trieste, V. Giorgieri 1, Trieste 34127, Italy; ‡ 535888 Fondazione Italiana Fegato ONLUS − Italian Liver Foundation NPO, Metabolic Liver Disease Unit, SS14 Km 163.5 Area Science Park Basovizza, Trieste 34149, Italy; § Materials Synthesis Department, 61790 Jožef Stefan Institute, Ljubljana 1000, Slovenia; ∥ Department of Pharmaceutical Technology, Faculty of Pharmacy, University of Ljubljana, Ljubljana 1000, Slovenia

## Abstract

5-Fluorouracil (5-FU) is a chemotherapeutic drug that is widely used to treat gastrointestinal cancers (e.g., hepatocellular carcinoma). Unfortunately, its systemic toxicity limits its clinical utility; therefore, new means for its targeted delivery at the pathological site are highly sought after to enhance therapeutic efficacy. In this work, we describe a 5-FU covalent conjugate with the self-assembling, heterochiral tripeptide ^D^Leu-Phe-Phe (lFF) via a redox-sensitive linker (5-FU-SS-lFF). The conjugate yields supramolecular hydrogels with rheological properties similar to those of lFF hydrogels and enables 5-FU release under reducing conditions, such as those present in the tumor microenvironment. We demonstrate the selectivity of the drug release under such conditions over a period of 24 h, with consequent significant cytotoxicity on cancer cells, as demonstrated on hepatocellular carcinoma spheroids. Overall, this study opens new opportunities for peptide-based supramolecular hydrogels as versatile and smart vehicles for the selective release of anticancer drugs under tumor-like reducing conditions.

## Graphical abstract

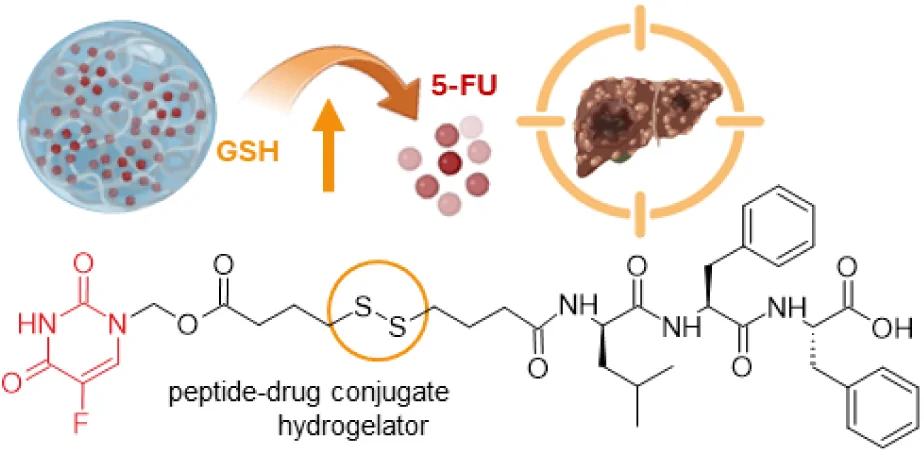

## 1 Introduction

Hydrogels are well-established and appealing biomaterials for biomedical uses, specifically when used as versatile drug delivery platforms.^1,2^ Hydrogels serve to encapsulate, dissolve, and protect therapeutics, allowing for favorable control over their release both in time and space.^3–5^ In this way, drug bioavailability and efficacy can be improved, while at the same time reducing toxicity due to high or uncontrolled dosages. Besides, hydrogels are often regarded as ideal materials in terms of biocompatibility, being composed of significant amounts of water and having viscoelastic properties similar to those of human tissues.^6^

Among the multitude of hydrogel classes that can be employed for drug delivery, those based on peptides have been gaining particular attention, especially due to their biocompatibility and biodegradability.^7–12^ Compared to traditional covalently cross-linked polymeric hydrogels, peptide-based gels rely on noncovalent interactions, such as hydrogen bonds, π–π stacking, hydrophobic and electrostatic interactions, leading to native-like, dynamic materials that can be formed and biodegraded *in vivo*. Specifically, supramolecular hydrogels based on ultrashort peptides are attractive vehicles for drug delivery, also because they can be produced easily and at low cost on a large scale.^13^ Short heterochiral peptides composed of d- and l-amino acids stack into β-sheets that fibrillate, eventually leading to the formation of 3-D networks and hydrogels. The combination of d- and l-building blocks in short sequences is quite useful, since it not only enables turn-like conformers that are prone to self-assembly but also provides some resistance against protease-mediated hydrolysis.^14^ One of the most popular examples of such short heterochiral peptides that is able to reliably form robust hydrogels under a variety of experimental conditions, including multicomponent systems, is given by ^D^Leu-Phe-Phe (lFF).^15–17^ This low-molecular-weight gelator has been previously used to noncovalently load and release various drugs, leading to sustained delivery over prolonged periods for hydrophobic molecules,^18^ but suffering from burst release of more hydrophilic ones, such as 5-fluorouracil (5-FU),^19^ thus limiting its applicability in cancer treatment.

The advantages offered by supramolecular, short peptide-based hydrogels as drug delivery systems are particularly useful for cancer treatment,^20,21^ since dosage, pharmacokinetics, and control of the release of chemotherapeutics are even more critical due to the severity of the adverse effects on quality of life. In particular, 5-fluorouracil (5-FU) is an antimetabolite that inhibits irreversibly thymidylate synthase, thereby halting DNA synthesis.^22^ It is commonly used in clinical practice for treating solid tumors, including gastrointestinal cancers such as gastric cancer, colorectal cancer, and hepatocellular carcinoma.^23–25^ However, its efficacy following systemic administration is compromised due to its short half-life, significant side effects, and susceptibility to drug resistance.^26^ To overcome some of these challenges, 5-FU has been incorporated into various hydrogel formulations. However, in such systems, where the drug is physically embedded, it is challenging to predict how the molecule will interact with the gelator component, resulting in variable loading efficiencies and release kinetics that can be difficult to control. In the case of lFF hydrogels, 5-FU did not interfere with the tripeptide self-assembly process, but it did not form significant and long-lasting interactions with the gelator molecules either, resulting in an uncontrolled and complete release within only a few hours.^19^ Therefore, a covalent approach may offer an alternative strategy, so long as hydrolysis can be tuned to match clinical needs, ideally triggered by the local microenvironment of the pathological site. For example, 5-FU can be covalently linked to a self-assembling short peptide, as demonstrated with Fmoc-Lys-Lys, via a hydrolyzable succinate group.^27^ However, self-assembly of the 5-FU-dipeptide conjugate into a supramolecular hydrogel shielded the hydrolyzable bonds from direct aqueous exposure, resulting in an extremely slow release of 5-FU (6% after over a month). While drug delivery could be sustained over long periods of time, it was primarily hydrolysis-dependent and was neither specific nor controlled to the extent typically required for chemotherapy applications in the clinic. For this reason, there is a clear need to design novel formulations based on biodegradable vehicles that fill this gap.

The incorporation of reduction-sensitive and cleavable disulfide linkages has been successfully employed in various formulations, ranging from nanoparticles to hydrogels, exploiting redox-active linkers or cysteine residues.^28–31^ This strategy has been used to prepare multifunctional anticancer prodrugs and to achieve tumor-specific release. Such systems can rapidly degrade and respond specifically to the tumor microenvironment, characterized by increased glutathione (GSH) concentrations (usually 1–10 mM, an order of magnitude higher than extracellular fluids and plasma^32–35^ ). We thus reasoned that an attractive covalent strategy for the controlled release of 5-FU from an lFF supramolecular hydrogel could use a disulfide linker, taking advantage of dithiodibutyric acid’s suitability for inclusion in the standard solid-phase peptide synthesis (SPPS) workflow. The resulting conjugate, 5-FU-SS-lFF, is composed of the anticancer drug 5-FU, a self-assembling heterochiral tripeptide lFF, and a redox-responsive disulfide linkage. We hypothesized that conjugation of 5-FU to lFF tripeptide through a disulfide-containing linker would not interfere with the tripeptide’s ability to self-assemble, while at the same time providing a redox-responsive mechanism for drug release, triggered by elevated GSH levels characteristic of tumor-like environments. To date, no peptide-based hydrogel formulations aimed at redox-triggered release of 5-FU have been reported. This work aims to investigate the drug-peptide conjugate’s capacity to self-assemble into a macroscopic hydrogel material that releases the drug in a controlled way, triggered by high concentrations of GSH. Finally, its anticancer properties are assessed on a relevant *in vitro* hepatocellular carcinoma model to demonstrate this system’s efficiency in cancer treatment as proof-of-concept.

## 2 Experimental Section

### 2.1 Materials

Unless stated otherwise, all reagents and solvents were purchased from Merck (Milan, Italy) and used without further purification. All Fmoc-protected amino acids and 2-chlorotrityl resin were obtained from Iris Biotech GmbH (Marktredwitz, Germany) and used as received. An in-line Millipore RiOs/Origin system provided Milli-Q water with a resistivity higher than 18 MΩ cm.

### 2.2 Synthesis

#### 2.2.1 Synthesis of 5-FU-DTBA Conjugate

5-FU (780.0 mg, 6.0 mmol) was first added to a 37% formaldehyde aqueous solution (1.3 mL), and the mixture was heated at 60 °C for 50 min. When the residue became fully transparent, excess water was removed under reduced pressure with anhydrous acetonitrile, yielding a transparent viscous oil. The residue was then dissolved in anhydrous THF (9.0 mL), followed by addition of dithiodibutyric acid (1430.0 mg, 6.0 mmol), DCC (743.0 mg, 3.6 mmol), and DMAP (148.0 mg, 1.2 mmol). The reaction mixture was stirred at RT for 24 h. The solids were removed by filtration, and the filtrate was concentrated under reduced pressure to yield a viscous yellow oil. The crude product was then thoroughly washed with 1 M NaHCO_3_, followed by acidification of the resulting aqueous solution (with concentrated HCl). The resulting white precipitate was separated by filtration, washed with water, and dried under high vacuum. The white solid was further purified by silica column chromatography (dry loading method), employing 1:1 EtOAc:cyclohexane supplemented with 0.5% v/v acetic acid. The final product (*R*_*f*_ = 0.2) was obtained as a white solid, with a yield of 18% relative to 5-FU. Purity and identity of the compound confirmed by NMR, ESI-MS, and analytical LC can be found in SI (Figures S1–S4).

#### 2.2.2 Peptide and Peptide–Drug Conjugate Synthesis

Tripeptide lFF was prepared by solid-phase peptide synthesis (SPPS), following an Fmoc-based strategy and using 2-chlorotrityl chloride resin (loading after the first amino acid coupling was 0.7 mmol/g). For all amino acids, amide coupling steps were accomplished by using Fmoc-amino acids, 1,3-diisopropylcarbodiimide (DIC), and Oxyma Pure B (300 mol % excess each relative to the resin) in DMF for 1.5 h. A piperidine solution (20% in DMF) was used to deprotect Fmoc groups for 2 × 5 min. After each coupling and deprotection step, the resin was thoroughly washed with DMF (3 × 2 min) and DCM (3 × 2 min). Peptide cleavage from the resin was accomplished by using DCM:TFA:H_2_O:TIPS (47.5:47.5:1:4) for 2 h, and the resulting solution was concentrated under compressed air until a dry yellowish residue was obtained.

For the synthesis of tripeptide–drug conjugate 5-FU-DTBA-lFF-OH (5-FU-SS-lFF), the same approach was used as described above. Following Fmoc removal from d-Leu, an additional amide coupling step was done: 5-FU-DTBA-COOH, DIC, and Oxyma Pure B (300 mol % excess each relative to the resin) in DMF were added to the resin and shaken for 2 h. The final drug–peptide conjugate was cleaved from the resin under the same conditions as described above and concentrated under air until dry crude product was obtained.

The crude lFF and 5-FU-SS-lFF were dissolved in a mixture of MeCN:H_2_O (30/70, 0.05% TFA), centrifuged at 15 000 rpm for 10 min, and filtered through a 0.2 μm filter before being purified via reversed-phase HPLC. Both lFF and 5-FU-SS-lFF were purified on an Agilent 1260 with a C-18 column (Kinetex, 5 μm, 100 Å, 250 × 10 mm, Phenomenex), *T* = 35 °C, flow = 3 mL min^–1^ with the following methods; for lFF, *t* = 0–2 min 30% MeCN (0.05% TFA), *t* = 16 min 95% MeCN (0.05% TFA), *t* = 16–18 min 95% MeCN (0.05% TFA), *R_t_* = 10.1 min; for 5-FU-SS-lFF, *t* = 0–2 min 30% MeCN (0.05% TFA), *t* = 16 min 65% MeCN (0.05% TFA), *t* = 19 min 95% MeCN (0.05% TFA), *t* = 19–21 min 95% MeCN (0.05% TFA), *R_t_* = 17.9 min. The collected fractions were then freeze-dried to yield the corresponding tripeptide lFF and 5-FU-SS-lFF conjugate, both as white powders. Samples’ purity was assessed by analytical LC, and their identity was confirmed by NMR and ESI-MS. The corresponding data can be found in SI (Figures S5–S6 for lFF, Figures S7–S12 for 5-FU-SS-lFF).

### 2.3 Self-Assembly and Hydrogel Preparation

Either tripeptide lFF or peptide–drug conjugate 5-FU-SS-lFF was first dissolved in sodium phosphate (0.1 M, pH 11.8) at double the desired concentration. Self-assembly, and thus gelation, was triggered by a pH switch. Specifically, an equal volume of mildly acidic sodium phosphate (0.1 M, pH 5.6) was added to the solution, bringing pH to 7.0 ± 0.1. Samples were then incubated at RT until hydrogels were fully formed. Unless otherwise specified, the final concentration for lFF and 5-FU-SS-lFF conjugate hydrogels was 5 mM.

### 2.4 Oscillatory Rheology

The viscoelastic properties of lFF and 5-FU-SS-lFF hydrogels were measured using an oscillatory Malvern Kinexus Ultra Plus Rheometer (Alfatest, Milan, Italy), which was set up with a 20 mm plate–plate parallel geometry and a solvent trap. All measurements were conducted at 25 °C (unless otherwise specified) by carefully preparing fresh hydrogels *in situ* on the measuring plate. For all formulations, the linear viscoelastic region (LVR) was identified by dynamic stress sweeps at an oscillation frequency of 1 Hz. Gelation kinetics were studied with time sweeps performed at a constant frequency of 1 Hz and a constant stress of 1 Pa. Dynamic frequency sweeps were carried out at a constant stress of 1 Pa in the frequency range of 0.01–10 Hz. The measurements were performed in triplicate and shown as mean ± standard deviation.

### 2.5 Circular Dichroism (CD)

Hydrogel samples were prepared at a 5 mM concentration directly in a 0.1 mm path-length sandwich-type quartz cell (Hellma Analytics) by placing 75 μL of solution B1 containing the peptide on the bottom coverslip of the sandwich cell, followed by adding 75 μL of buffer B2, before placing the top coverslip to sandwich the solution. The cell was then placed in a cell holder, and the measurements were acquired on a Jasco J815 Spectropolarimeter (Jasco Europe, Cremella, Italy). Spectra were recorded with 1 s integrations, 15 accumulations, and a step size of 1 nm with a bandwidth of 1 nm over a range of wavelengths from 190 to 260 nm at 25 °C (using a Peltier as the temperature controller).

### 2.6 Fourier-Transform Infrared Spectroscopy (FT-IR)

FT-IR spectra were recorded at RT on a PerkinElmer Spectrum Two spectrometer, equipped with an attenuated total reflectance (ATR) sampling accessory. Hydrogel samples were dried under vacuum overnight prior to measurements, and spectra were recorded in the range from 400 to 4000 cm^–1^ with a resolution of 4 cm^–1^.

### 2.7 Transmission Electron Microscopy (TEM)

TEM images were acquired on a JEOL JEM 2100 (Japan) instrument operating at 100 kV. TEM grids (copper-grid-supported carbon film) were first exposed to the UV-ozone cleaner (UV-Ozone ProCleaner Plus) for 5 min before hydrogel samples (prepared the day before) were delicately deposited on top of them. Next, the deposited hydrogels were allowed to dry for 15 min at RT prior to adding a contrast aqueous tungsten phosphate solution (pH 7.4).

### 2.8 LC–MS Analysis

LC–MS analyses were performed using an analytical Agilent 1260 Infinity system (Luna, 5 μm, 100 Å, 150 × 2 mm, Phenomenex) at a flow rate of 0.3 mL/min, with a quadrupole ESI-MS detector, by dissolving samples in a 1:1 mixture of MeCN/water with 0.1% formic acid. The following gradients were used: *lFF*: *t* = 0 min, 5% MeCN; *t* = 20 min, 95% MeCN, *R_t_* = 15.6 min; *5-FU-DTBA* conjugate: *t* = 0–2 min, 5% MeCN; *t* = 22 min, 95% MeCN; *t* = 22–29 min, 95% MeCN, *R_t_* = 19.1 min; *5-FU-SS-lFF*: *t* = 0–2 min, 5% MeCN; *t* = 22 min, 95% MeCN; *t* = 22–27 min, 95% MeCN, *R*_*t*_ = 23.4 min; drug release studies: *t* = 0–2 min, 5% MeCN; *t* = 22 min, 90% MeCN; *t* = 26 min, 92% MeCN; *t* = 30 min, 95% MeCN; *t* = 30–34 min, 95% MeCN, *R_t_* (drug–peptide conjugate) = 23.3 min, *R_t_* (byproduct) = 23.1 min.

### 2.9 Nuclear Magnetic Resonance (NMR)

The NMR spectra were recorded on a Varian Innova Instrument (Agilent Technologies, Milan, Italy). ^1^H NMR and ^13^C NMR spectra were recorded at 400 and 100 MHz, respectively, with chemical shifts reported as δ in parts per million (ppm), referenced against the residual solvent peak. Data were analyzed using MestReNova software (version 14.2.1).

### 2.10 TFA Counterion Exchange with Chloride Ions and ^19^F-NMR

A total of 5.0 mg of 5-FU-SS-lFF was dissolved in Milli-Q water/acetonitrile 10/2 (1.0 mg/mL), and 200 μL of a 100 mM solution of HCl were added. The solution was gently vortexed and sonicated at RT for 10 min to allow counterion exchange. The solution was lyophilized overnight, and the resulting powder was reanalyzed by 400 MHz (DMSO-*d*_6_) (Figure S17 in SI). The same procedure was adopted for the lFF tripeptide (NMR data not shown). The procedure was previously reported by Bellotto et al.^36^

### 2.11 Hydrogel Stability

In preweighed vials, 150 μL of either lFF or 5-FU-SS-lFF hydrogels (at 5 mM) were prepared as described in Section 2.3. Hydrogel stability was assessed by a gravimetric method under both physiological and reducing conditions. After pH-triggered self-assembly, the hydrogels were incubated overnight to equilibrate, and the initial weight was recorded (*W*_0_). Then, a total of 1.0 mL of either PBS (pH 7.4) or glutathione (GSH) solution (5 mM in PBS) was added, and the samples were incubated at 37 °C. At predetermined time points, the medium was carefully removed, and hydrogel weight was measured (*W*_*t*_), followed by replacement with fresh medium. The swelling ratio (defined as *W*_*t*_/*W*_0_) was used to monitor hydrogel swelling and/or degradation properties over time. All tested samples and conditions were measured in triplicate.

### 2.12 Drug Release Studies

Hydrogels based on 5-FU-SS-lFF were prepared in glass vials (total volume of 150 μL, at 5 mM) following the method described in Section 2.3. To allow for complete gelation, samples were incubated at RT overnight. The next day, 1.0 mL of either PBS (0.1 M, pH 7.4) or GSH (5 mM in PBS) was added to mimic physiological and tumor-like reducing conditions, respectively, and samples were incubated at 37 °C with shaking at 60 rpm. At the indicated time points, the samples were supplemented with 3 μL of formic acid to lower pH and quench GSH activity, followed by gentle stirring and manual manipulation to completely disintegrate the remaining hydrogels. Resulting solutions were analyzed by LC–MS (as described in Section 2.8), and areas under the curve (AUC) of the cleaved byproduct (SH-lFF) and native gelator molecule (5-FU-SS-lFF) were calculated relative to the highest AUC. For SH-lFF, the highest AUC was taken at time point 24 h, whereas the highest AUC for 5-FU-SS-lFF was taken at time point zero. Relative release quantification of 5-FU was therefore indirectly determined as the AUC ratio of the byproduct SH-lFF following GSH treatment.

### 2.13 Cell Culture and Spheroid Generation

Human liver cancer spheroids were set up using the HepG2 cell line. Cells were cultured in high-glucose Dulbecco’s Modified Eagle Medium (DMEM) supplemented with 10% fetal bovine serum, 100 U/mL penicillin/streptomycin, and 2 mM l-glutamine at 37 °C and 5% (v/v) CO_2_. All cell culture reagents were obtained from Euroclone (Milan, Italy). To generate liver cancer spheroids, HepG2 cells were seeded at a concentration of 5000 cells/mL on CELLSTAR culture 96-well ultralow attachment (ULA) plates (Greiner Bio-One, Frickenhausen, Germany) for 96 h under gentle shaking, similar to what was described previously.^37^ Spheroids were imaged using a Nikon Eclipse TS100 microscope (Nikon, Tokyo, Japan) equipped with a 10× objective.

Human colorectal cancer spheroids were set up using the Caco-2 cell line, whereas the MDCK II cell line was used to generate spheroids as a nontumoral epithelial control. Both Caco-2 and MDCK II spheroids were generated and cultured by following the same method described for HepG2 spheroids.

### 2.14 Spheroid Exposure to 5-FU-Loaded Hydrogel-Conditioned Media

Hydrogels were prepared at 5 mM (total volume of 200 μL), as described in section 2.3. To obtain 5-FU-SS-lFF hydrogel-conditioned media, complete cell culture medium was added to each hydrogel preparation (a total of 1.8 mL), with or without 5 mM GSH. Hydrogels were then incubated for 24 h at 37 °C, and the conditioned medium was collected, passed through a 0.2 μm pore-size filter, and used immediately to treat liver cancer spheroids. Drug-free hydrogels (lFF) were also prepared to be used as a control condition. Liver cancer spheroids, colorectal cancer spheroids, and MDCK II spheroids were exposed to conditioned media from all hydrogel preparations, as well as to additional control conditions, including 5 mM GSH and 0.5 mM 5-FU. The duration of exposure for all conditions was 24 h.

### 2.15 Cell Viability

Cell viability was determined by measuring the total adenosine triphosphate (ATP) content using the CellTiter-Glo 3D Cell Viability Assay (G9682, Promega, Milan, Italy), according to the manufacturer’s instructions. In brief, 70 μL of cell culture medium was removed from each well, and 30 μL of CellTiter-Glo 3D reagent was added. The spheroids were then lysed by vigorous pipetting. After a 25 min incubation at RT in the dark, 50 μL of the resulting lysate was transferred to a white 1/2 Area OptiPlate 96-well plate (PerkinElmer, Waltham, MA, USA), and the bioluminescence signal was measured using an Enspire Multimode Plate Reader (PerkinElmer, Waltham, MA, USA). A minimum of three spheroids per treatment condition was used to measure total ATP levels. Data were corrected for the background, normalized to untreated cells, and presented as relative cell viability.

### 2.16 Data Analysis and Statistics

All data are presented as mean ± standard deviation (SD), unless stated otherwise. Statistical analysis was performed using one-way analysis of variance (ANOVA), followed by Tukey’s multiple comparison test. A *p*-value <0.05 was considered significant. GraphPad Prism (version 10.1.1; GraphPad Software Inc., La Jolla, CA, USA) was used for statistical analysis. All experiments were repeated independently at least three times.

### 2.17 Safety Statement

No unexpected or unusually high safety hazards were encountered.

## 3 Results and Discussion

### 3.1 Synthesis of 5-FU-SS-lFF Conjugate

The synthetic design was based on using a redox-responsive disulfide linker that undergoes cleavage under reducing conditions, thereby releasing 5-FU in a tumor-like environment. In this way, 5-FU can be covalently attached to the tripeptide hydrogelator even after self-assembly into a hydrogel, to then be released upon exposure to GSH in the tumor microenvironment (Figure 1). To this end, dithiodibutyric acid (DTBA) was used as a disulfide-containing bifunctional linker.^38^ The synthetic overview is given in Scheme 1A. 5-FU was first reacted with aqueous formaldehyde to quantitatively obtain 1,3-bis­(hydroxymethyl)-5-fluorouracil, following a previously reported procedure.^39,40^ The DTBA linker was then coupled to 5-FU via a direct esterification reaction at room temperature, using DCC as a coupling agent and DMAP as a catalyst, yielding a disulfide-containing intermediate building block (5-FU-DTBA, 18% yield). The obtained intermediate was mainly *N* ^1^-substituted 5-FU. It has been previously reported that following the reaction between 5-FU and formaldehyde, the bis-alkylated intermediate is formed, but the reaction workup leads to *N* ^1^-hydroxymethyl 5-FU as the main product due to *N* ^3^-hydroxymethyl instability and ease of hydrolysis, leaving the free NH group on 5-FU.^27,39^ At this point, 5-FU-DTBA had still one carboxyl group available for coupling to the amine of the peptide N-terminus. The identity of 5-FU-DTBA was successfully confirmed by ^1^H NMR, ^13^C NMR, and LC–MS analyses (Figures S1–S4). The synthetic yield suggests the need for future optimization of industrial applications and scalability.

**Figure 1:**
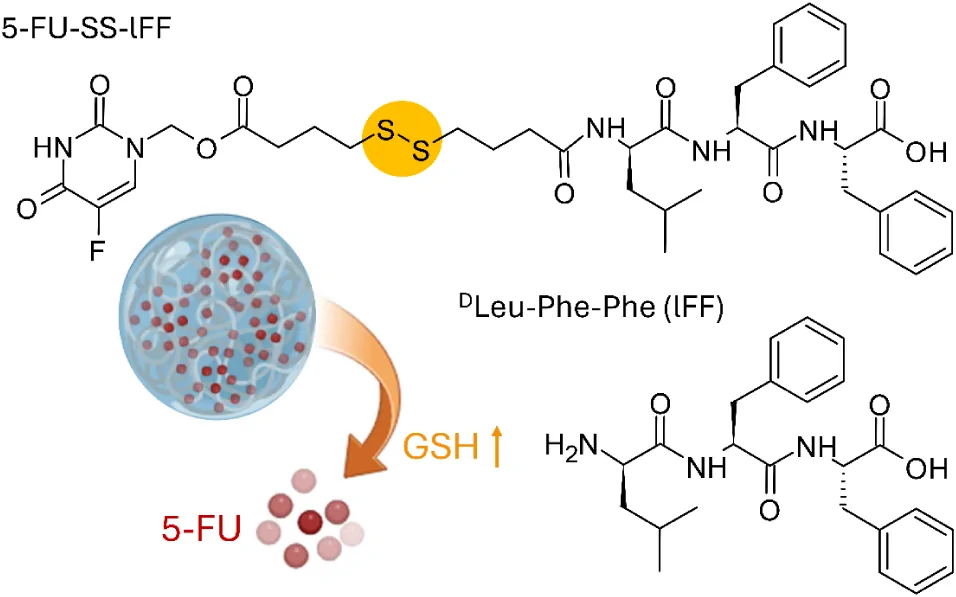
Design of the redox-sensitive system. Schematic representation of the hydrogelator molecules, 5-FU-SS-lFF conjugate (top) and lFF tripeptide (bottom), and their self-assembly into a hydrogel that releases 5-FU upon increased concentrations of glutathione (GSH).

**Scheme 1:**
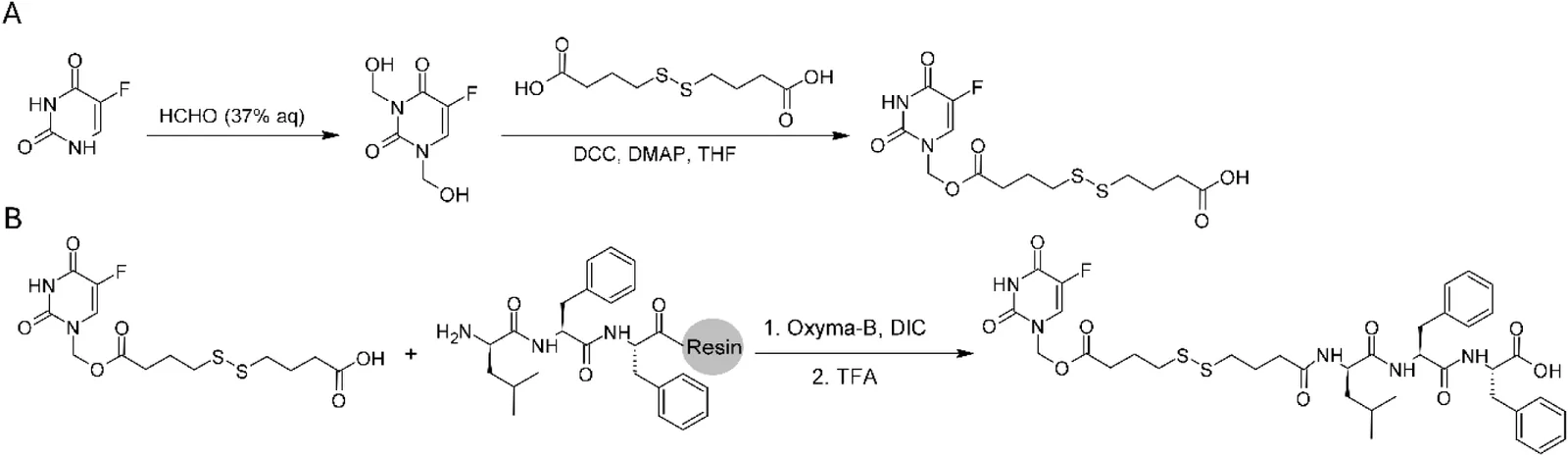
Synthesis Route for Functionalization of 5-FU with Dithiodibutyric Acid (A); Conjugation of 5-FU-DTBA Intermediate to the Peptide Chain during SPPS, Followed by 5-FU-SS-lFF Cleavage from the Resin (B)

The heterochiral tripeptide lFF hydrogelator was prepared by standard Fmoc-based solid-phase peptide synthesis (SPPS), as previously reported.^19^ The peptide structure was successfully identified by ^1^H NMR, ^13^C NMR, and LC–MS (Figures S5–S6), and the data were in agreement with the previously reported findings.^19^ Next, on-the-resin conjugation of the 5-FU-DTBA linker was employed to obtain the desired 5-FU-SS-lFF conjugate. Specifically, the 5-FU-DTBA intermediate was coupled to the Fmoc-deprotected N-terminus of the leucine residue during SPPS (Scheme 1B). The coupling was performed under the same conditions used for all amino acids (3× molar excess of the linker to the peptide content), and the cleavage from the resin was performed using a TFA/DCM/H_2_O/TIPS cocktail, as for lFF. The heterochiral tripeptide serves as a carrier for 5-FU, which upon disulfide bond cleavage under reducing conditions would release the drug. The 5-FU-SS-lFF conjugate was obtained through preparative HPLC, with a conjugation yield of 83%, and its structure was verified by ^1^H NMR, ^13^C NMR, and LC–MS analyses (Figures S7–S12).

### 3.2 Hydrogel Formation and Physicochemical Characterization

To assess the capacity of lFF and 5-FU-SS-lFF to self-assemble into hydrogels, a well-established procedure was employed.^17,19^ Namely, self-assembly occurred in phosphate buffer following a pH switch from alkaline to neutral at a final concentration of 5 mM. As expected, lFF formed rapidly a macroscopic hydrogel (Figure 2A), in line with previous reports.^17,19^ To our delight, despite being devoid of a free amino group at the N-terminus (at physiological pH, 5-FU possesses no net charge), 5-FU-SS-lFF also formed a self-supporting hydrogel (Figure 2A), as can be seen from the vial inversion test, albeit with slower kinetics (vide infra).

**Figure 2:**
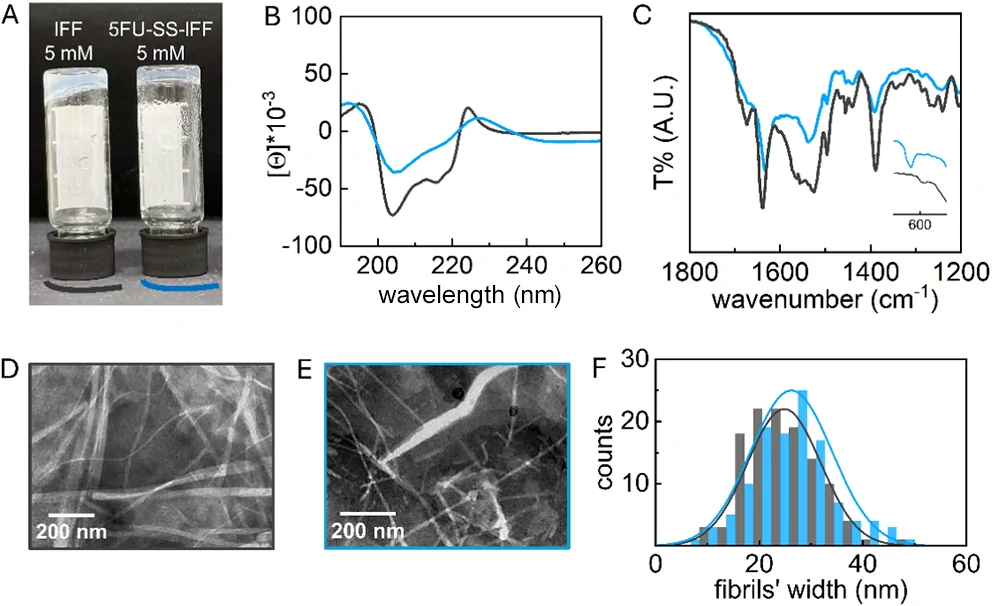
Physicochemical characterization of hydrogels. Hydrogels from lFF and 5-FU-SS-lFF at 5 mM during the inverted vial test (A); circular dichroism (CD) spectra of lFF and 5-FU-SS-lFF hydrogels at 5 mM (B); FT-IR spectra of lFF and 5-FU-SS-lFF (C), inset 650–550 cm^–1^; TEM image of lFF at 5 mM (D); TEM image of 5-FU-SS-lFF at 5 mM (E); fiber size distribution from TEM analyses (F). Dark gray and blue colors in images correspond to lFF and 5-FU-SS-lFF samples, respectively.

To gain insights into conformational changes leading to the self-assembly of 5-FU-SS-lFF compared to lFF, circular dichroism (CD) spectra were recorded on 5 mM hydrogels. In line with previous reports,^19^ lFF displayed prominent negative signals in the region of 200–220 nm (minima at 204 and 215 nm), indicative of β-structure formation and fibrillation (Figure 2B). The observed negative signal is compatible with stacking into supramolecular β-structures of l-chirality, as observed before with other self-assembling tripeptides of the same stereoconfiguration.^14^ However, somewhat less pronounced and broader negative peaks observed in the same region for 5-FU-SS-lFF likely indicate the presence of mixed conformations, including β-structures (Figure 2B). This difference was ascribed to packing defects caused by the linker molecule and 5-FU during the self-assembly process of the lFF counterpart. In CD spectra with 5-FU physically embedded, the CD signal of lFF was virtually unaffected by the presence of drug molecules, suggesting that self-assembly could be less homogeneous and ordered for 5-FU-SS-lFF.^19^

FT-IR spectra also confirmed the presence of secondary structure signatures. Both spectra were dominated by band frequencies typical of β structures—amide I band frequencies. These were detected at 1638 and 1632 cm^–1^ for lFF and 5-FU-SS-lFF, respectively. Amide II band frequencies were observed at 1526 cm^–1^ for lFF and at 1537 cm^–1^ for 5-FU-SS-lFF (Figure 2C), suggesting that in both gels, there was the presence of secondary structures and a hydrogen-bonding network. Besides studying hydrogel conformation, FT-IR revealed a structural feature typical for 5-FU-SS-lFF, a C–S stretching band at 618 cm^–1^ (Figure 2C, inset), not observed in lFF.

We were pleased to observe that both samples displayed ordered nanostructure formation when imaged with TEM. The nanomorphology analysis revealed that lFF and 5-FU-SS-lFF peptides assembled into fibrils (Figure 2D–E, respectively) with comparable size distributions, i.e., 24.7 ± 7.1 nm for lFF and 26.2 ± 7.9 nm for 5-FU-SS-lFF (*n* = 100). In both cases, the fibrils tended to run parallel to each other, occasionally bundling into larger fibers, as previously reported for lFF-based supramolecular systems.^18,19^

In summary, CD, FT-IR, and TEM data showed that the covalent attachment of 5-FU to lFF *via* a linker did not impede the self-assembly into gelling nanofibrils.

### 3.3 Hydrogel Gelation and Viscoelastic Characterization

The gelation kinetics and viscoelastic properties of the hydrogels were assessed by oscillatory rheometry. Both samples were tested at a 5 mM final concentration at pH 7.4. Figure 3A shows the rapid gelation of lFF, with an immediate onset upon reaching neutral pH (when the measurement started) and being completed within 10 min. The storage modulus (*G*′) at the plateau was 5.7 ± 0.55 kPa, which is consistent with previously reported *G*′ values (∼10 kPa at 10 mM).^19^ In contrast, gelation kinetics of 5-FU-SS-lFF was significantly slower, as first observed with the vial inversion test (Section 3.2). The gelation process started immediately upon reaching neutral pH, but the overall gelation process was gradual and slow, taking several hours to reach a plateau (Figure 3D). After 4 h, the hydrogel displayed a *G*′ of 4.5 ± 1.8 kPa, around 20% lower than its lFF counterpart. This observation indicated that despite being able to efficiently self-assemble into a gel, the modified structure of 5-FU-SS-lFF likely resulted in supramolecular packing defects, consistent with CD data analysis (Section 3.2). The absence of a positive charge in this gelator, in contrast with the zwitterionic lFF, could also be responsible for a softer gel due to the inability to establish the typical ionic bridges between the gelator termini, as typically observed for this type of hydrogelator. In comparison, the lFF hydrogel with physically incorporated 5-FU displayed unaffected gelation kinetics relative to the peptide alone. However, it did possess a slightly increased storage modulus that was ascribed to additional physical interactions between lFF fibrils and 5-FU molecules.^19^

**Figure 3:**
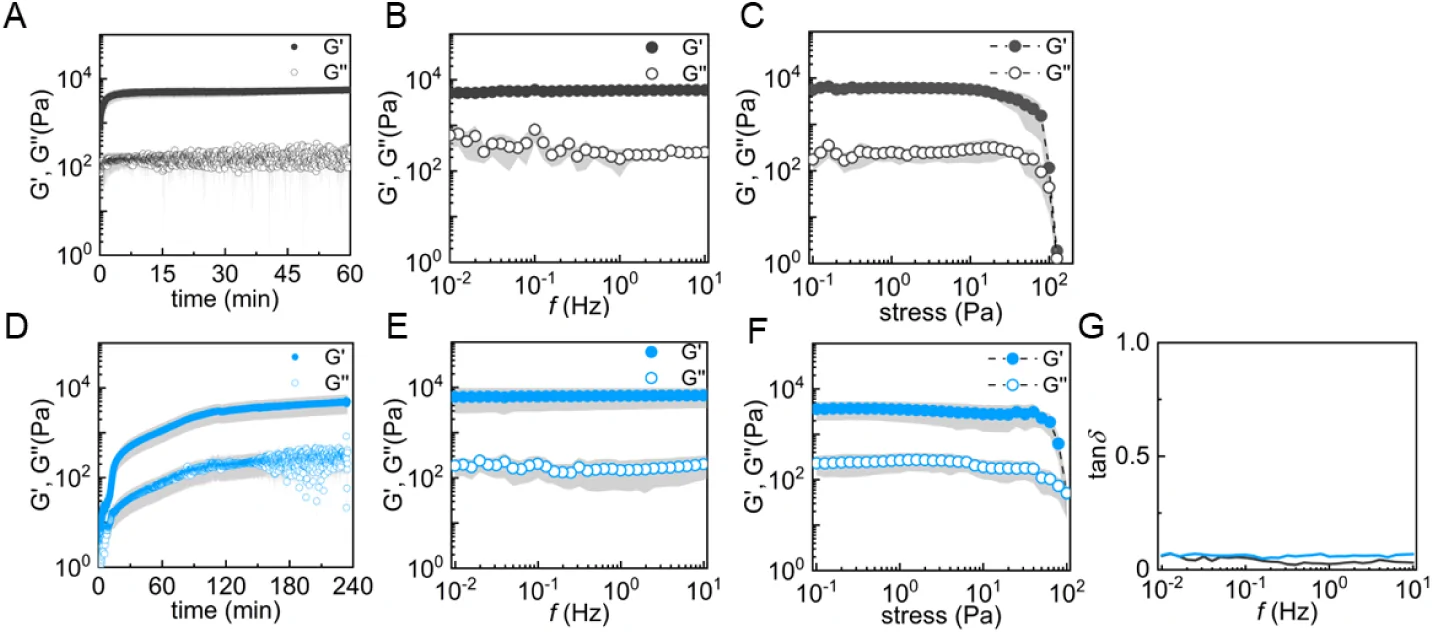
Rheological characterization of hydrogels. Gelation kinetics (time sweep) of lFF hydrogel at 5 mM (*f* = 1 Hz, σ = 1 Pa) (A); frequency sweep of lFF hydrogel (*f* = 0.01–10 Hz, σ = 1 Pa) (B); stress sweep for lFF hydrogel (*f* = 1 Hz, σ = 0.1–100 Pa) (C); time sweep for 5-FU-SS-lFF hydrogel at 5 mM (*f* = 1 Hz, σ = 1 Pa) (D); frequency sweep for 5-FU-SS-lFF hydrogel (*f* = 0.01–10 Hz, σ = 1 Pa) (E); stress sweep for 5-FU-SS-lFF hydrogel (*f* = 1 Hz, σ = 0.1–100 Pa) (F); loss factor (*G*″/*G*′) for lFF and 5-FU-SS-lFF hydrogels at 5 mM, as determined from the corresponding frequency sweep measurements (G). Dark gray and blue colors in images correspond to lFF and 5-FU-SS-lFF samples, respectively. The measurements were performed in triplicate and shown as mean ± standard deviation.

Next, the frequency sweep test revealed that both hydrogels exhibited predominantly elastic-like behavior, with *G*′ and *G*″ being independent of the applied frequency, and *G*′ > *G*″ over the entire frequency range that was examined (Figure 3B and E). Such elastic behavior indicated that there were strong interfibrillar interactions, with no significant structural rearrangement or relaxation processes in the network occurring during the probed time scale. The same conclusion can be drawn by looking at the loss factor tan δ (*G*″/*G*′) as a function of the applied frequency. In fact, tan δ was independent of the frequency, ranging between 0.02 and 0.05 (Figure 3G). This constant tan δ suggests an elastic character of the tested materials, with limited capacity for dissipating elastic energy.^41,42^

The stress sweep analysis showed that the gels fluidized at increasing stresses, with a decrease of both *G*′ and *G*″ (Figure 3C and F). In the presence of covalently attached 5-FU, the gel’s resistance to shear stress was lower by almost 25% relative to the lFF hydrogel. In fact, the yield stress (determined as the stress at which *G*′ deviates by 5% from the LVR) was found to be 39 ± 7.8 Pa for 5-FU-SS-lFF and 43 ± 4.3 Pa for the lFF hydrogel. The observed yield stresses were of the same order of magnitude as those found for lFF at 10 mM, with or without 5-FU physically loaded^19^ (55 and 80 Pa, respectively).

Overall, viscoelastic characterization of the gels revealed that the covalent attachment of 5-FU to lFF hydrogelator slowed the gelation and reduced the storage modulus and yield stress of the resulting hydrogel. However, the overall viscoelastic profiles were found to be elastic-like, comparable between the two gels, and in line with previous reports on similar hydrogelator systems. Ongoing work is focused on optimizing the formulation and fabrication approaches toward expanding materials’ processability.

### 3.4 *In Vitro* Release Studies

The release of 5-FU from 5-FU-SS-lFF hydrogels was studied in tumor-resembling, reducing conditions (PBS 0.1 M, pH 7.4, GSH at 5 mM) and under physiological conditions (PBS 0.1 M, pH 7.4) (Figure 4A and C). PBS supplemented with GSH was selected to provide a controlled reducing environment for evaluating the contribution of GSH-mediated disulfide bond cleavage to drug release, as widely used in studies assessing reduction-triggered drug release systems.^43–45^ Although biological fluids are more physiologically relevant, their complex composition may introduce additional factors that influence release independently of the reduction-responsive mechanism, and future studies will be directed toward providing additional insight into performance of the drug delivery system in the presence of complex biological media. The release was studied with LC–MS, by monitoring the depletion of starting hydrogelator 5-FU-SS-lFF and the increase of the side product resulting from the disulfide bond cleavage, i.e., SH-lFF (lFF tripeptide with half of the linker remaining) (Figure 4B). Monitoring directly the production of 5-FU-SH with the available setup was challenging because of the overlap in the chromatogram between the corresponding peak and those of GSH and GSSG. However, there is a reliable and known stoichiometric relationship between SH-lFF and 5-FU-SH, considering that the generation of SH-lFF is directly related to the generation of the prodrug 5-FU-SH, as they are the only two possible products from the disulfide cleavage. Similar indirect methods for monitoring drug release have been previously employed in disulfide-based prodrug systems.^46–49^ In addition, a calibration-free method, as employed here for quantification, that relies on AUC ratio determination was previously used in a similar system for anticancer peptide release from polymeric nanoparticles.^45^ It is worth noting that the employed method does present limitations, as it only considers the release due to disulfide bond cleavage and not other possible degradation mechanisms that could lead to drug release.

**Figure 4:**
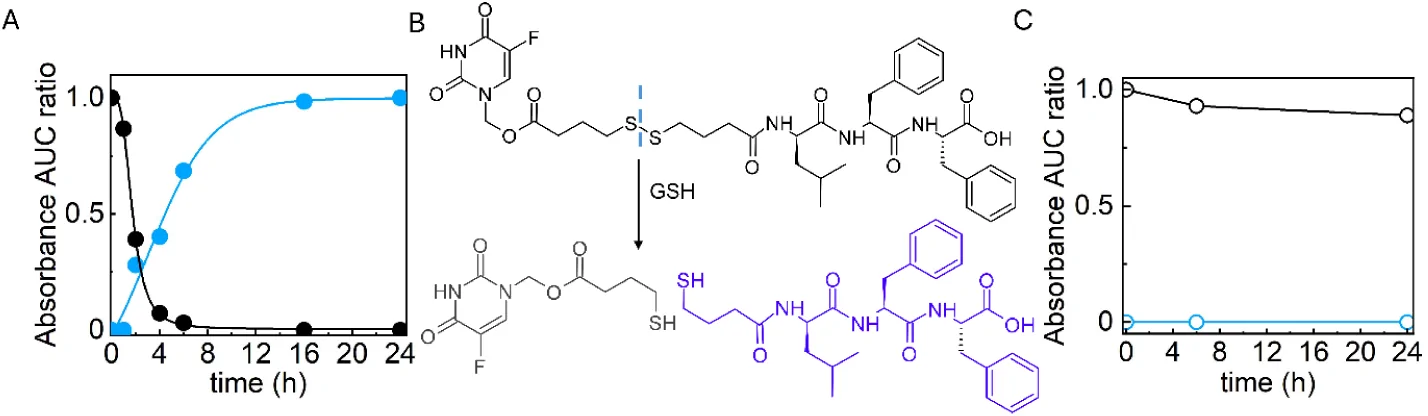
Drug release studies. Release kinetics (expressed as the AUC ratio to the maximal recorded absorbance) of 5-FU-SH determined indirectly via lFF-SH release under reducing conditions (5 mM GSH, PBS pH 7.4, 37 °C) (A); the hydrogelator molecule 5-FU-SS-lFF undergoes disulfide bond cleavage to release lFF-SH and 5-FU-SH intermediates (B); release kinetics of 5-FU-SS-lFF in the absence of GSH (PBS pH 7.4, 37 °C) (C); dark gray and blue colors in the images correspond to the 5-FU-SS-lFF starting hydrogelator molecule and SH-lFF, respectively. Figures S13–S14 illustrate chromatograms used for AUC ratio determination.

Figure 4A shows the release profiles at 24 h for 5-FU-SS-lFF and SH-lFF. Specifically, there was an increasing trend in SH-lFF (and consequently of 5-FU-SH) release during the first 12 h, reaching full release after 16 h (see Figures S13 and S14 for relative chromatograms). The identity of the monitored species was confirmed by ESI-MS (see Figures S15 and S16). The observed release kinetics were not very fast, as opposed to some other redox-sensitive systems, such as nanogels and nanoparticles,^45,50^ in which a complete release took only 60 min. We inferred that the disulfide bonds in the present system were not as exposed to the aqueous environment for rapid cleavage but were rather somewhat less accessible and protected by hydrophobic regions within the supramolecular assembly. Nevertheless, full release was observed after a period of 16–24 h. At the same time, a decrease in the number of starting 5-FU-SS-lFF molecules was detected, indicating their consumption as the cleavage progressed (Figure 4A). The more rapid depletion of 5-FU-SS-lFF compared with SH-lFF generation suggests the presence of other mechanisms that could contribute to a minor extent to its degradation, most likely involving the hydrolytic cleavage of the ester moiety. Indeed, such ester hydrolysis was deliberately used for the slow and sustained release of 5-FU from Fmoc-dipeptide-based self-assembled hydrogels.^27,51^

The release profiles in the absence of GSH showed that no generation of SH-lFF was detected during 24 h, with ∼10% decrease in 5-FU-SS-lFF (Figure 4C). These observations confirmed that no disulfide bond cleavage took place in the absence of GSH, and any loss of 5-FU-SS-lFF came from other, considerably slower mechanisms, such as ester hydrolysis. If the release profiles between the present system and the one with physically loaded 5-FU in lFF are compared, in 5-FU-SS-lFF, the drug remained stable in the gel (unless triggered by reducing conditions), whereas in the latter system the drug was fully and rapidly released, reaching a plateau after only 8 h.^19^ Therefore, covalently linking the drug to the lFF hydrogelator molecule significantly improved the release profile, allowing for a triggered and specific release.

It is also worth noting that in the present system, 5-FU forms part of the hydrogelator molecular structure; therefore stoichiometrically, the drug loading is fixed. In our specific case, the drug loading was 16.6% and, accordingly, the hydrogel contained 0.06 wt % of 5-FU. The amount of the drug can thus be easily tailored by adjusting the final hydrogelator concentration. This is an advantage compared to the lFF gel with physically loaded 5-FU, where drug loading highly depends on both the drug concentration and that of lFF and on the physical interactions between lFF and 5-FU molecules during self-assembly. Furthermore, compared to the lFF system with physically loaded 5-FU, covalently linking the drug to the lFF hydrogelator molecule suppresses the burst release while allowing for a redox-triggered release. Owing to the mo lecular design of the system, each gelator molecule carries a single covalently attached drug molecule, resulting in a fixed drug loading that cannot be independently tuned as in conventional carrier-based hydrogels. Although the drug content of the hydrated gel is relatively low (≈0.06 wt %), this reflects the high water content of the hydrogel, ensuring homogeneous drug incorporation throughout the hydrogel network. Importantly, the delivered dose scales directly with gel concentration and can therefore be increased by increasing the hydrogel concentration without modifying the underlying molecular design. While the current formulation provides a lower absolute drug payload than conventional injectable formulations of 5-FU (25–50 mg/mL),^52^ the therapeutic strategy here is based on prolonged retention at the tumor site and controlled release of 5-FU following reduction of the disulfide linker by the elevated glutathione levels characteristic of the intracellular tumor environment. This localized, stimuli-responsive release is expected to maximize drug exposure within the tumor while minimizing premature release and systemic toxicity. Further studies are needed for optimization and assessment of drug payload and therapeutic dosing.

To prove whether the released species containing 5-FU retained their chemotherapeutic efficacy, *in vitro* anticancer tests were conducted, as discussed in Section 3.6.

### 3.5 Hydrogel Stability

Since we noticed that these hydrogels could be easily manually disassembled after being treated with GSH (release studies, section 3.4), we went further to investigate hydrogels’ stability over time. Similar heterochiral tripeptide-based hydrogels have mostly been assessed for their thermoreversibility, where gels are disassembled after being exposed to heating and then allowed to reform upon cooling to RT.^53,54^ However, their stability over time at a fixed temperature has not been explored. Here, we monitored their weight over time under physiological conditions (pH 7.4, 37 °C), with or without GSH (5 mM). The ratio between the weight at designated time points and the starting weight was expressed as a swelling ratio, reflecting the hydrogel’s capacity to swell or erode over time. As previously reported, the swelling ratio is commonly taken as a measure of hydrogel stability over time.^41,44^ Both 5-FU-SS-lFF and lFF gels were prepared at a 5 mM final concentration, and a total of 1 mL of medium was added (PBS with or without GSH 5 mM). Figure 5A displays the variation in the hydrogels’ weight during the first 6 h following the addition of the medium. Strikingly, there was no swelling over time for either of the two hydrogels; rather a decrease in weight was observed. Most likely, this was because these gels were formed purely by physical interactions between small building blocks, so unlike charged polymer-based gels that tend to swell over time, these supramolecular gels when immersed in water tend to degrade without a swelling phase. Specifically, the lFF hydrogel degraded a bit slower in the first hours than 5-FU-SS-lFF, with a swelling ratio reaching ∼0.8 after 6 h, regardless of the presence of GSH. This suggests that the presence of GSH in the solution did not significantly alter the self-assembled structure of lFF, nor did it promote faster disassembly (Figure 5A, gray full symbols). In contrast, the 5-FU-SS-lFF hydrogel was more responsive to the presence of GSH. Namely, degradation was visibly steeper in the first hours, with a swelling ratio reaching ∼0.55 after 6 h (Figure 5A, blue full symbols). This observation was attributed to the cleavage of the disulfide bonds and therefore, partial loss of a structural component of the self-assembling system. In comparison, without GSH, the stability of 5-FU-SS-lFF was quite similar to that of lFF hydrogels (Figure 5A, blue open symbols).

**Figure 5:**
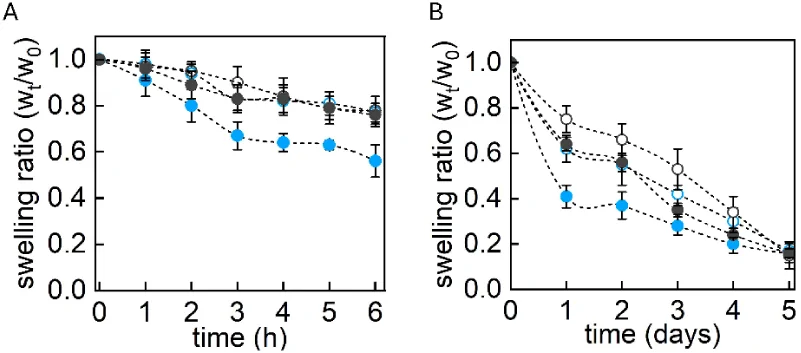
Hydrogel stability over time. Swelling ratio as a function of time (hours) for 5-FU-SS-lFF and lFF hydrogels at 5 mM, pH 7.4, *T* = 37 °C (A); swelling ratio as a function of time (days) (B); dark gray and blue colors in images correspond to lFF and 5-FU-SS-lFF samples, respectively. Open symbols correspond to PBS only; full symbols correspond to PBS supplemented with 5 mM GSH.

The stability of the hydrogels was also monitored in the following days, and it was observed that surprisingly, all formulations degraded by day 5, although at different rates. Specifically, lFF without GSH displayed the most gradual degradation, whereas the steepest rate was observed for 5-FU-SS-lFF in the presence of GSH. The major mass loss (over 50%) for 5-FU-SS-lFF after 24 h indicated that drug release took place, confirming observations found in the release studies (Section 3.4). Regardless of the differences in swelling ratio at intermediate time points, all hydrogels converged by day 5, when they completely disassembled. We attribute this limited hydrogel stability over time to their supramolecular character and lack of covalent cross-linking points in the network, as has been observed for other low-molecular-weight supramolecular hydrogels.^55^ However, for the intended final application of this study, limited hydrogel stability does not pose a significant problem, as the release of 5-FU is designed to be rapid and complete under reducing conditions. In addition, the stability study showcased an excellent degradability profile for these heterochiral tripeptide-based supramolecular hydrogels, which is directly linked to their functionality and safety.

### 3.6 *In Vitro* Anticancer Activity

Since 5-FU is indicated in the treatment of hepatocellular carcinoma and other solid gastrointestinal cancers, to assess the anticancer activity of the released species containing 5-FU from the 5-FU-SS-lFF hydrogels, an *in vitro* spheroid model of human hepatocellular carcinoma was employed. Human hepatocellular carcinoma spheroids were successfully generated by using HepG2 cells cultured on ULA plates for 72 h. To assess cell viability after 24 h of treatment with 5-FU-SS-lFF hydrogels, the ATP assay was performed as previously described.^37^ The 5-FU-SS-lFF and lFF hydrogels were prepared as described in Section 3.2 at a 5 mM concentration and incubated in cell culture medium supplemented with 5 mM GSH for 24 h to allow drug release, as reported in Section 3.4. The conditioned media were collected after that time and used to treat spheroids for 24 h. Untreated spheroids were used as controls. The cell viability results are shown in Figure 6A, while Figure 6B displays representative bright-field images of each treatment condition. Compared to untreated spheroids, lFF hydrogels, either with or without GSH, did not significantly reduce viability, indicating that the carrier used in this study, lFF hydrogel itself, does not induce cytotoxicity at concentrations used to formulate hydrogels. Bright-field images confirm that the morphology of spheroids exposed to lFF hydrogels was comparable to the control condition (Figure 6B).

**Figure 6:**
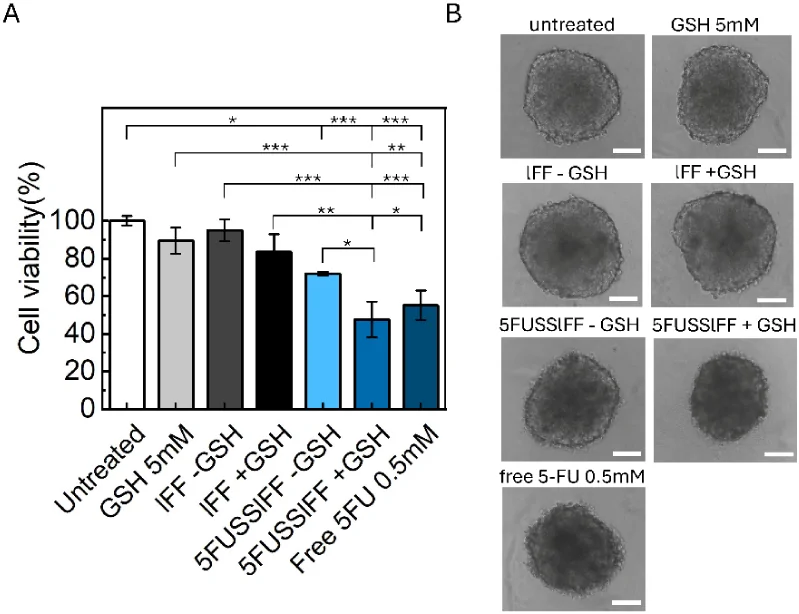
Anticancer effect of hydrogel formulations. The anticancer effect of lFF and 5-FU-SS-lFF hydrogels under physiological and reducing conditions (in the presence of 5 mM GSH) in human liver cancer spheroids, assessed by ATP quantification and determination of cell viability, following 24 h exposure to conditioned media from hydrogel preparations. Untreated spheroids were used as a control, and spheroids treated with 5 mM GSH were used to assess the effect of GSH alone. Free 5-FU at 0.5 mM was used as a positive control (A). Representative bright-field images of liver spheroids after 24 h treatment for all tested conditions (as indicated in the images); scale bar = 100 μm (B). Data are derived from three independent experiments performed in triplicate and expressed as mean ± SEM. *p < 0.05, **p < 0.01, ***p < 0.001 (1-way ANOVA followed by Dunnett’s multiple comparison test, using untreated cells as a control).

Noteworthily, prior to cell viability experiments, both lFF and 5-FU-SS-lFF were treated to remove trifluoroacetate (TFA) ions, which are known to be cytotoxic^56^ and are usually present in the sample as counterions after HPLC purification (mobile phase supplemented with TFA). The method used for exchanging TFA ions with chloride ions was previously reported^36^ (see Figure S17 for ^19^F NMR before and after TFA removal). Therefore, the observed effects on cells in this study could be exerted only by the materials themselves and not by possible cytotoxic contaminants.

In conclusion, the formulations based on 5-FU-SS-lFF under reducing conditions (with GSH) significantly reduced cell viability by 52% after 24 h of treatment. In contrast, the 5-FU-SS-lFF formulation in the absence of GSH caused a reduction of cell viability by 28%. In the latter case, a somewhat compromised cell viability can be ascribed to ester hydrolysis-mediated 5-FU release.^27,51^ Conversely, GSH-mediated disulfide cleavage induced a significant reduction in cell viability, confirming the anticancer activity of the 5-FU-SS-lFF mediated by redox-triggered release. To assess whether the observed anticancer effect from 5-FU-SS-lFF (prodrug) under reducing conditions corresponds to the free drug, free 5-FU was used as a positive control; free 5-FU was used at 0.5 mM, a concentration that reflects the maximum theoretical concentration of the drug present in the hydrogel preparation (5 mM in hydrogel, subsequently diluted 10 times by the release medium). In this case, free 5-FU caused a reduction in cell viability by 45%, which is in line with the effect observed for the 5-FU-SS-lFF hydrogel. Therefore, the species containing 5-FU that are released from the gel in the presence of GSH retained the inherent cytotoxic effect of the equivalent concentration of the free 5-FU. Following disulfide bond cleavage, the release of free 5-FU takes place following hydrolytic cleavage of the acyloxymethyl moiety, as reported with similar 5-FU derivatives.^27,57–59^ In addition, representative bright-field images of spheroids treated with 5-FU-SS-lFF and free 5-FU clearly showed spheroid shrinkage and cell death. Finally, GSH at a 5 mM concentration had a minimal impact on cell viability, thus suggesting that the effects seen from each formulation could be ascribed exclusively to the species released from the hydrogels.

In addition, considering that 5-FU is used in treating several gastrointestinal cancers, another model was used to assess the anticancer activity of the present delivery platform. Specifically, human colorectal cancer spheroids based on the Caco-2 cell line were subjected to the same treatment as HepG2 spheroids. The results shown in Figure S18 indicate that the same observations and trends seen for liver cancer spheroids are also noticed with the colorectal cancer model. Namely, a significant reduction in cell viability was observed in the case of the GSH-treated 5-FU-SS-lFF hydrogel (37%), comparable to the free drug control (41%). These data suggest the overall efficacy of the present prodrug hydrogelator system across different gastrointestinal cancers, reinforcing its potential applicability in treating these conditions.

Lastly, the healthy cell control, given by MDCK II-based nontumoral spheroids, was used to assess overall cytotoxicity on healthy cells and the selectivity of the system. The results shown in Figure S19 suggest that MDCK II spheroids displayed good cytocompatibility with the delivery platform in the absence of triggered release. However, moderate cytotoxicity seen once the hydrogel was treated with GSH suggests the need for optimization of the formulation to enhance selectivity and minimize side effects on healthy cells. It should be noted that the employed experimental setup, based on conditioned release media, as also seen in other studies,^44^ is not very indicative of overall selectivity and systemic toxicity, for which future *in vivo* studies are required.

In summary, this study demonstrates the efficient drug release and anticancer activity of the 5-FU-SS-lFF system and its potential for effective cancer therapy. The heterochiral self-assembling tripeptide with a conjugated anticancer drug allows for a triggered drug release with uncompromised activity. Therefore, such a controlled-release platform, along with tailored drug loading, could improve 5-FU efficacy and reduce its dosage-dependent toxicity, as well as undesired side effects in physiological sites beyond the pathological target site.

## 4 Conclusions

Self-organized supramolecular hydrogels based on heterochiral tripeptides represent an extremely versatile class of soft materials suitable for biomedical applications such as the delivery of chemotherapeutic agents. In the present study, we evaluated whether the lFF hydrogelator could be used to form drug delivery systems upon covalent modification with the chemotherapeutic drug 5-FU *via* a redox-sensitive dithiodibutyric acid linker. Our results demonstrate that covalent attachment of 5-FU to lFF yields a 5-FU-SS-lFF conjugate that retains self-assembling capacity comparable to that of native lFF. Rheological studies revealed that 5-FU-SS-lFF hydrogels required longer self-assembly times, resulting in gels with slightly lower stiffness. These differences can be likely attributed to defects in supramolecular packing. However, once fully formed, both lFF and 5-FU-SS-lFF hydrogels exhibited similar viscoelastic and stability properties. Under a reducing tumor microenvironment, the conjugate-based hydrogels underwent efficient disulfide bond cleavage, releasing 5-FU in a controlled manner. *In vitro* studies further demonstrated that 5-FU released from the hydrogels exerted cytotoxic effects on both human hepatocellular carcinoma and human colorectal cancer spheroids, comparable to those of free 5-FU.

We therefore propose that carefully designed hydrogels based on self-assembling heterochiral short peptides hold significant promise as versatile and stimuli-responsive drug delivery platforms, particularly given their ability to directly incorporate drug molecules into the hydrogelator structure, thereby enabling controlled drug loading and release.

## Supplementary Material



## Notes

NMR spectroscopy and analytical LC data for 5-FU-DTBA intermediate; NMR spectroscopy and analytical LC data for lFF tripeptide; NMR spectroscopy and analytical LC data for 5-FU-SS-lFF conjugate; LC–MS data for drug release studies; ^19^F-NMR of 5-FU-SS-lFF before and after removal of TFA counterion; cytotoxicity data on Caco-2 and MDCK II spheroids (PDF)
