# Supplementary material for: Tumor-Responsive Delivery of 5‑Fluorouracil from a Redox-Triggered Supramolecular Gelator
Source: Bioconjug Chem. 2026 Aug 10;37(8):1571–81. doi: 10.1021/acs.bioconjchem.6c00108 (PMC13495549; doi:10.1021/acs.bioconjchem.6c00108)
Supplement: Supplementary file 1 [file bc6c00108_si_001.pdf]

Tumor-responsive delivery of 5-fluorouracil from a redox-triggered supramolecular gelator

Marko Mihajlovic,[a]* Milos Mihajlovic,[b] Paola Alletto,[a] Slavko Kralj,[cd] Natalia Rosso [b] and Silvia Marchesan [a]*

**Corresponding authors: marko.mihajlovic@units.it, smarchesan@units.it*

a. Department of Chemical and Pharmaceutical Sciences, University of Trieste, V. Giorgieri 1, 34127 Trieste, Italy.

b. Fondazione Italiana Fegato ONLUS – Italian Liver Foundation NPO, Metabolic Liver Disease Unit, SS14 Km 163.5 Area Science Park Basovizza, 34149 Trieste, Italy.

c. Materials Synthesis Department, Jožef Stefan Institute, 1000 Ljubljana, Slovenia

d. Department of Pharmaceutical Technology, Faculty of Pharmacy, University of Ljubljana, 1000 Ljubljana, Slovenia

Table of Contents

S1. Spectroscopic and analytical LC data for 5-FU-DTBA intermediate……………….................2

S2. Spectroscopic and analytical LC data for lFF tripeptide….…….……………………………….4

S3. Spectroscopic and analytical LC data for 5-FU-SS-lFF conjugate…….………………………..6

S4. LC-MS data for drug release studies………………………………………………………….....10

S5. ^{19}F-NMR of 5-FU-SS-lFF before and after removal of TFA counterion.…………………………..12

S6. Cytotoxicity test using a colorectal cancer spheroid model…………………………………………13

S7. Cytotoxicity test using a non-tumoral cell spheroid model…………………………………………….14

S1. Spectroscopic and analytical LC data for 5-FU-DTBA (NMR, ESI-MS and LC)

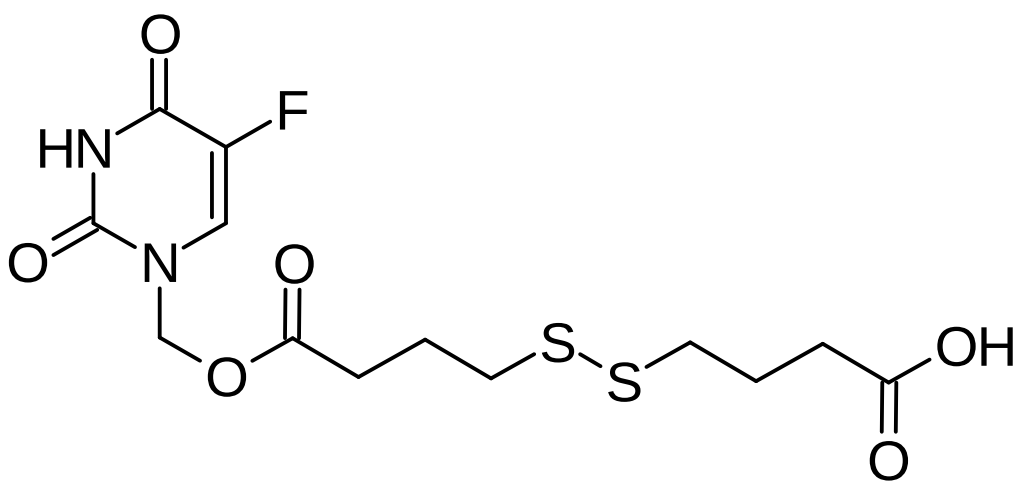

^{1}H-NMR (400 MHz, CD_3OD): δ 7.96 (d, *J* = 6.1 Hz, 1H), 5.68 (s, 2H), 2.77 (m, 4H), 2.55 (t, *J* = 7.2 Hz, 2H), 2.45 (t, *J* = 7.3 Hz, 2H), 2.04 (m, 4H); **^{13}C-NMR** (400 MHz, CD_3OD): δ 175.4, 172.8, 158.3, 149.7, 138.8, 129.3, 70.3, 37.1, 37.0, 31.8, 31.6, 24.0, 23.6. **MS (ESI)** m/z 403.0 $[M+Na]^+$ $C_{13}H_{17}FN_2O_6S_2$ requires 380.1.

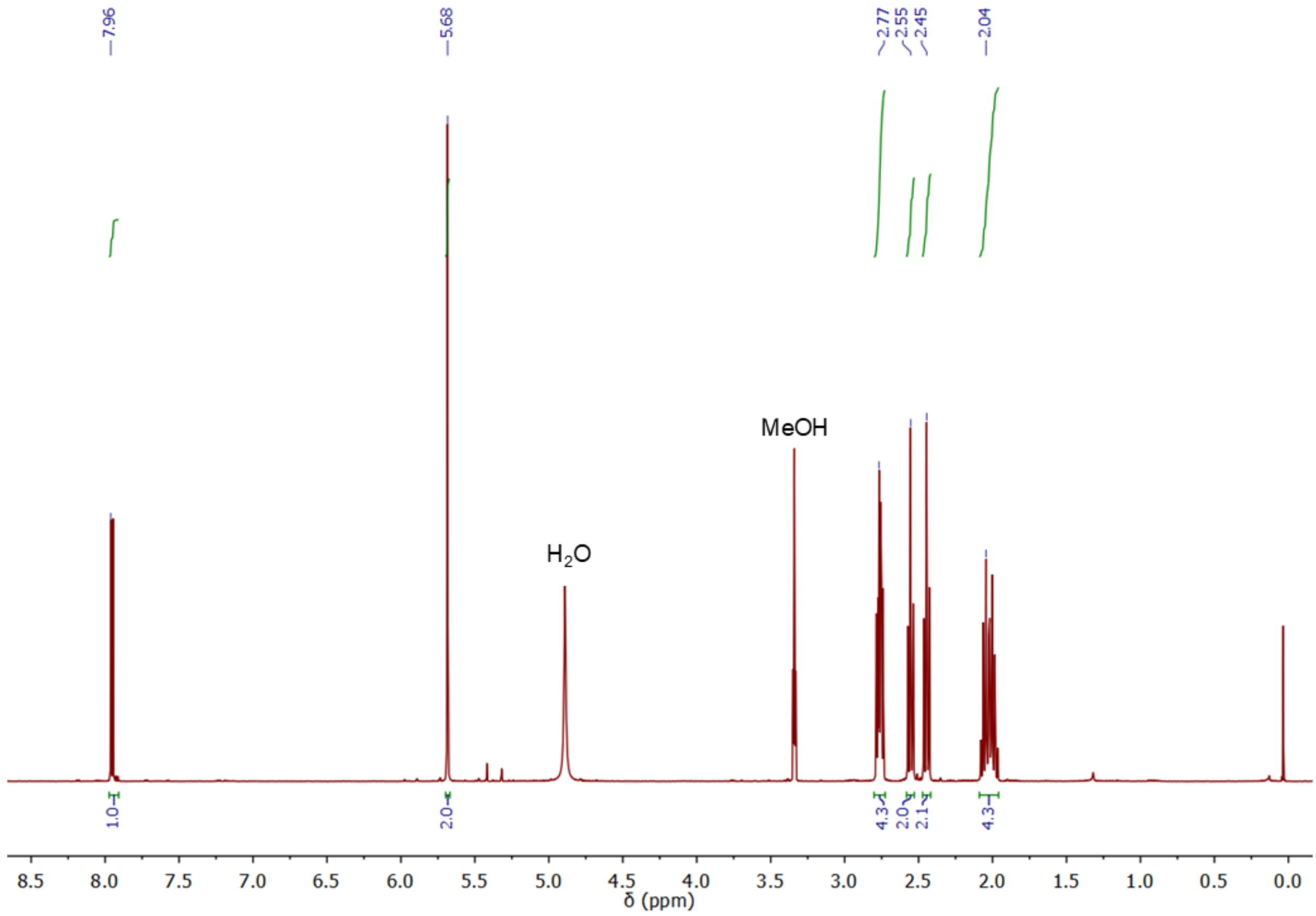

Figure S1. ^{1}H-NMR spectrum of 5-FU-DTBA intermediate.

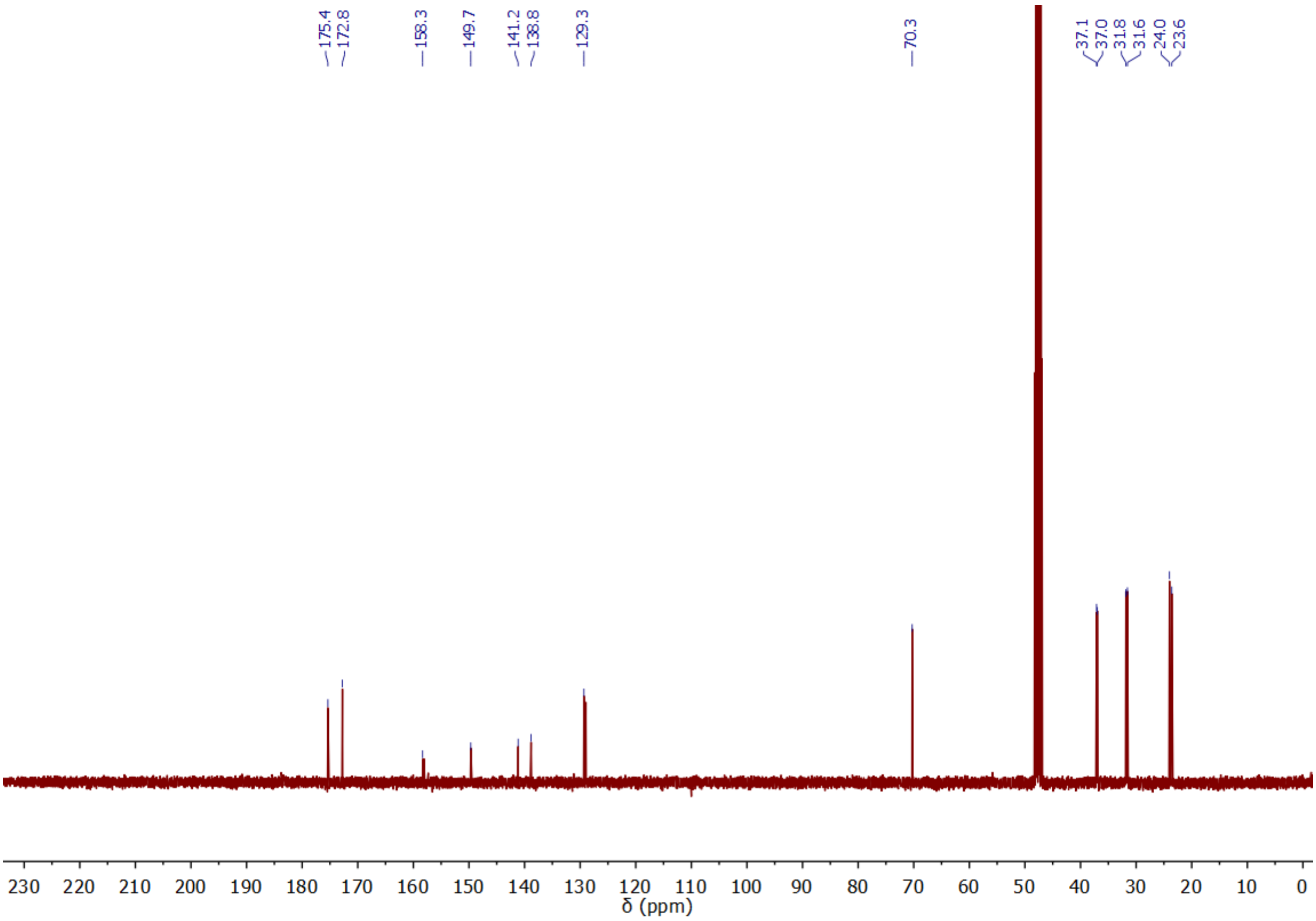

Figure S2. ^{13}C-NMR spectrum of 5-FU-DTBA intermediate.

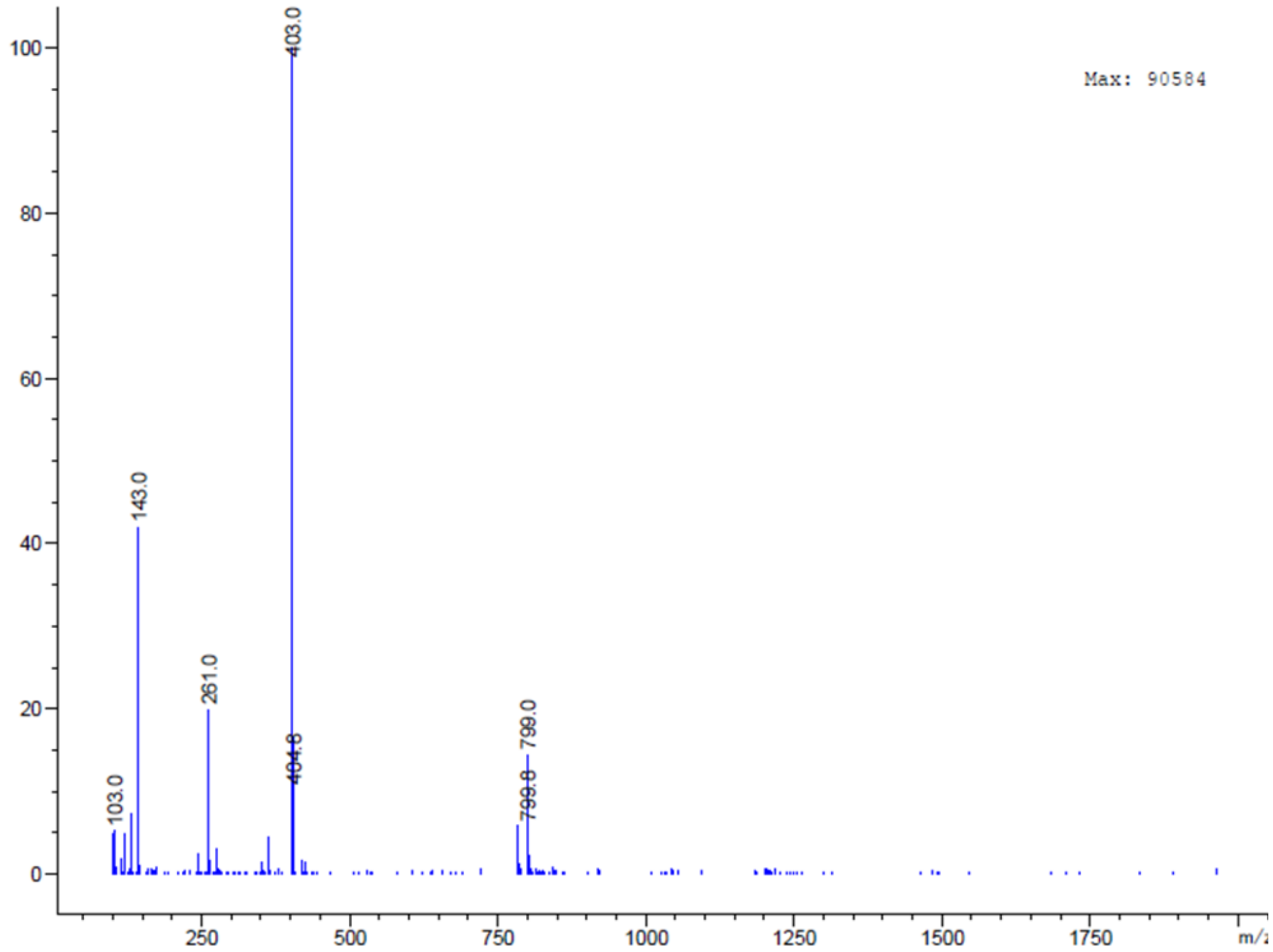

Figure S3. ESI-MS spectrum (positive ion mode) of 5-FU-DTBA intermediate.

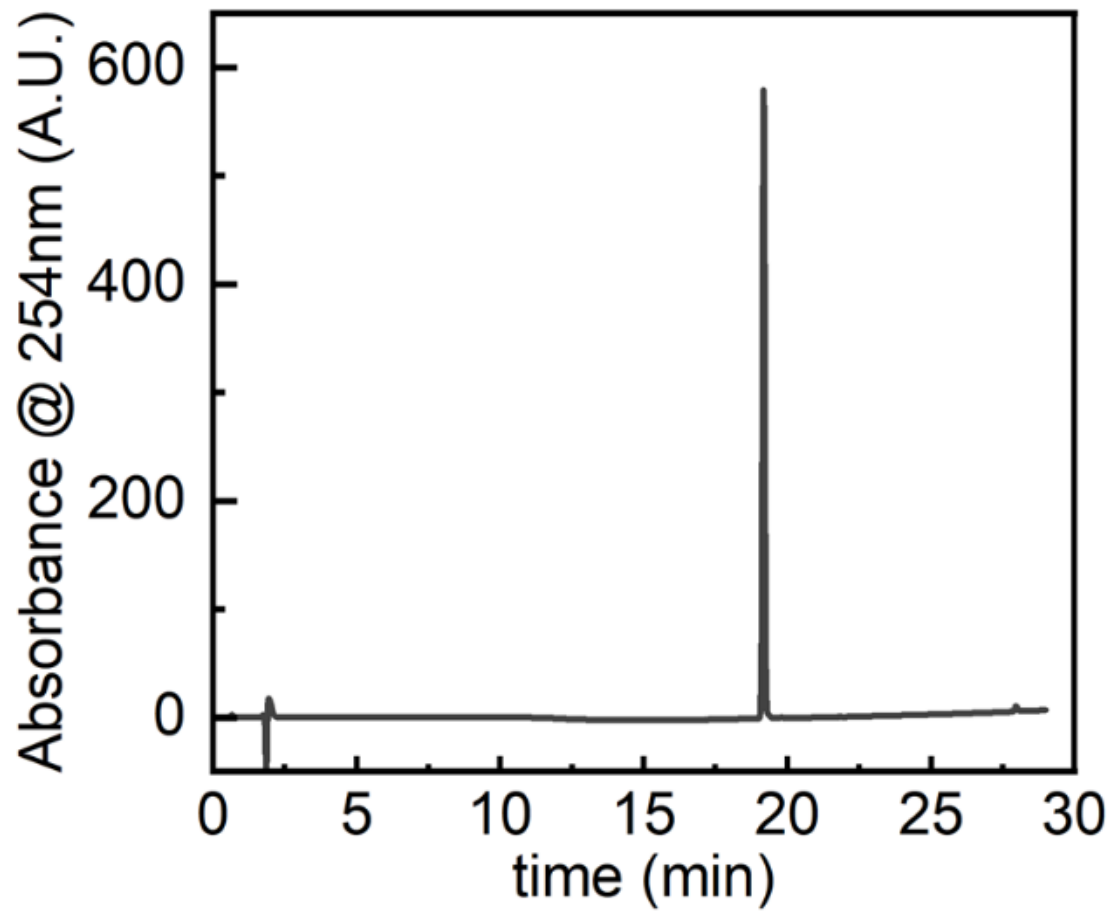

Figure S4. Analytical LC trace for 5-FU-DTBA intermediate. Retention time (R_t): 19.2 min.

S2. Spectroscopic and analytical LC data for lFF (NMR, ESI-MS and LC)

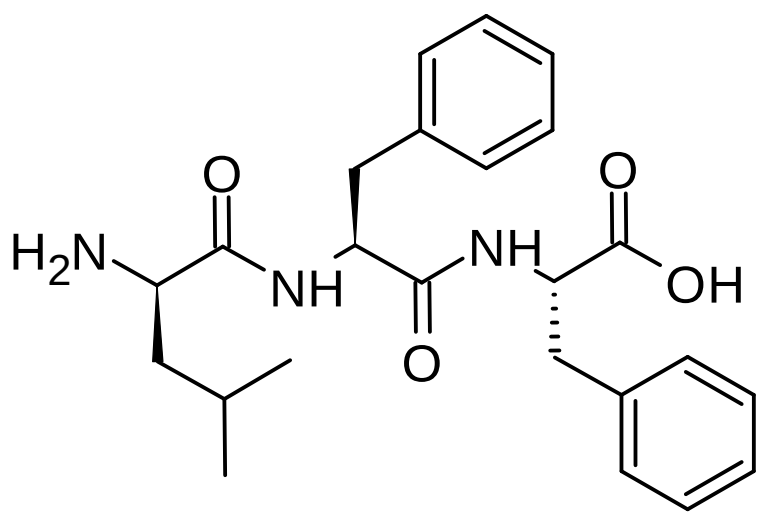

The NMR spectroscopic data for lFF tripeptide are in line with values reported in literature, as published in Parisi E, Garcia AM, Marson D, Posocco P, Marchesan S. Gels. 2019 Jan 26;5(1):5, and not reported here: **^{1}H-NMR** (400 MHz, DMSO-*d_6*): δ (ppm) 8.74 (d, J = 8 Hz, 1H, NH), 8.60 (d, J = 7.8 Hz, 1H, NH), 7.35- 7.15 (m, 10H, Ar), 4.71 (m, 1H, *α*CH), 4.47 (m, 1H, *α*CH), 3.63 (m, J = 6.9 Hz 1H, *α*CH), 3.10 (m, 2H, *β*CH), 2.95 (dd, 1H, J = 14.0, 9.2 Hz, *β*CH_2), 2.66 (dd, J = 13.8; 11.4 Hz, 1H, *β*CH_2), 1.20-1.05 (m, 3H, *β*CH_2 and *γ*CH), 0.68 (d, 6H, *δ*CH_3); **^{13}C-NMR** (100 MHz, DMSO-*d_6*): δ (ppm) 172.7, 171.2, 168.7 (3 x CO); 137.5, 137.4, 129.3, 129.1, 128.3, 128.0, 126.5, 126.3 (10 x Ar); 53.7, 53.6, 50.6, (3 x *α*C); 40.3 (1 x CH), 38.1, 36.6 (2 x *β*CH_2); 23.1 (*γ*CH) 22.5, 2 21.7 (2 x *δ*CH_3). **ESI-MS** m/z 426.0 $[M+H]^+$ $C_{24}H_{31}N_3O_4$ requires 425.2.

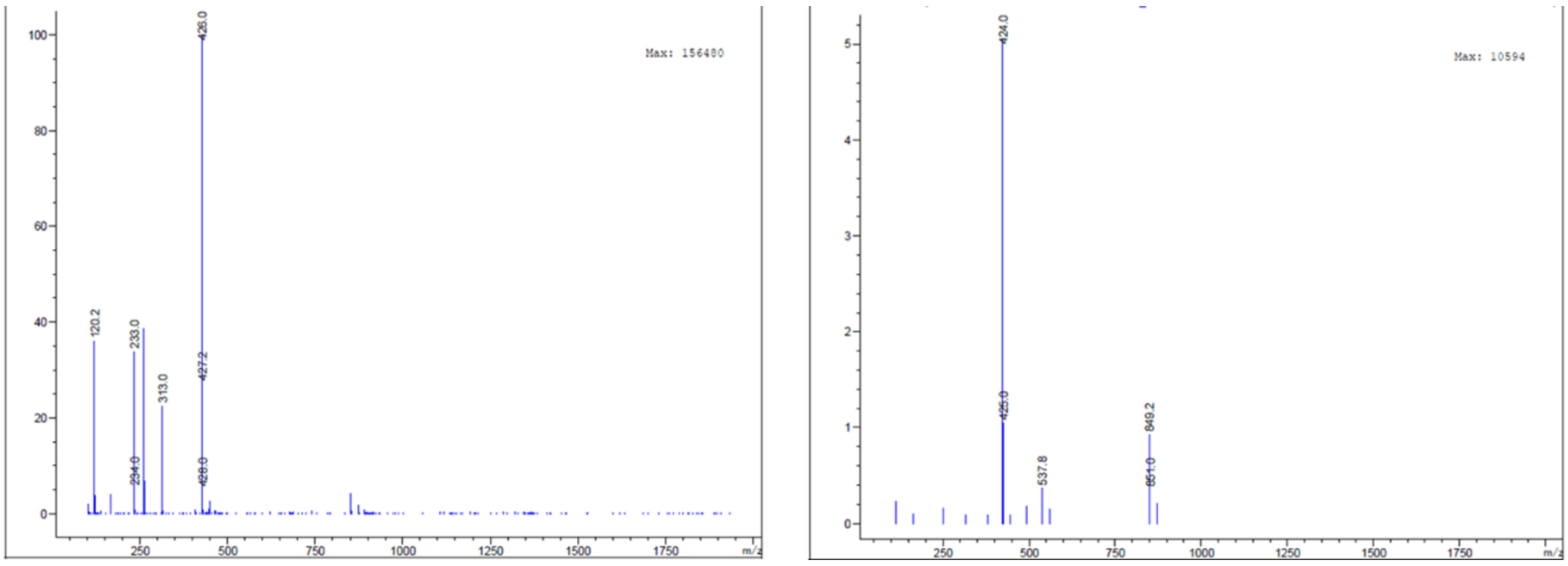

Figure S5. ESI-MS spectrum (positive and negative ion modes) for lFF.

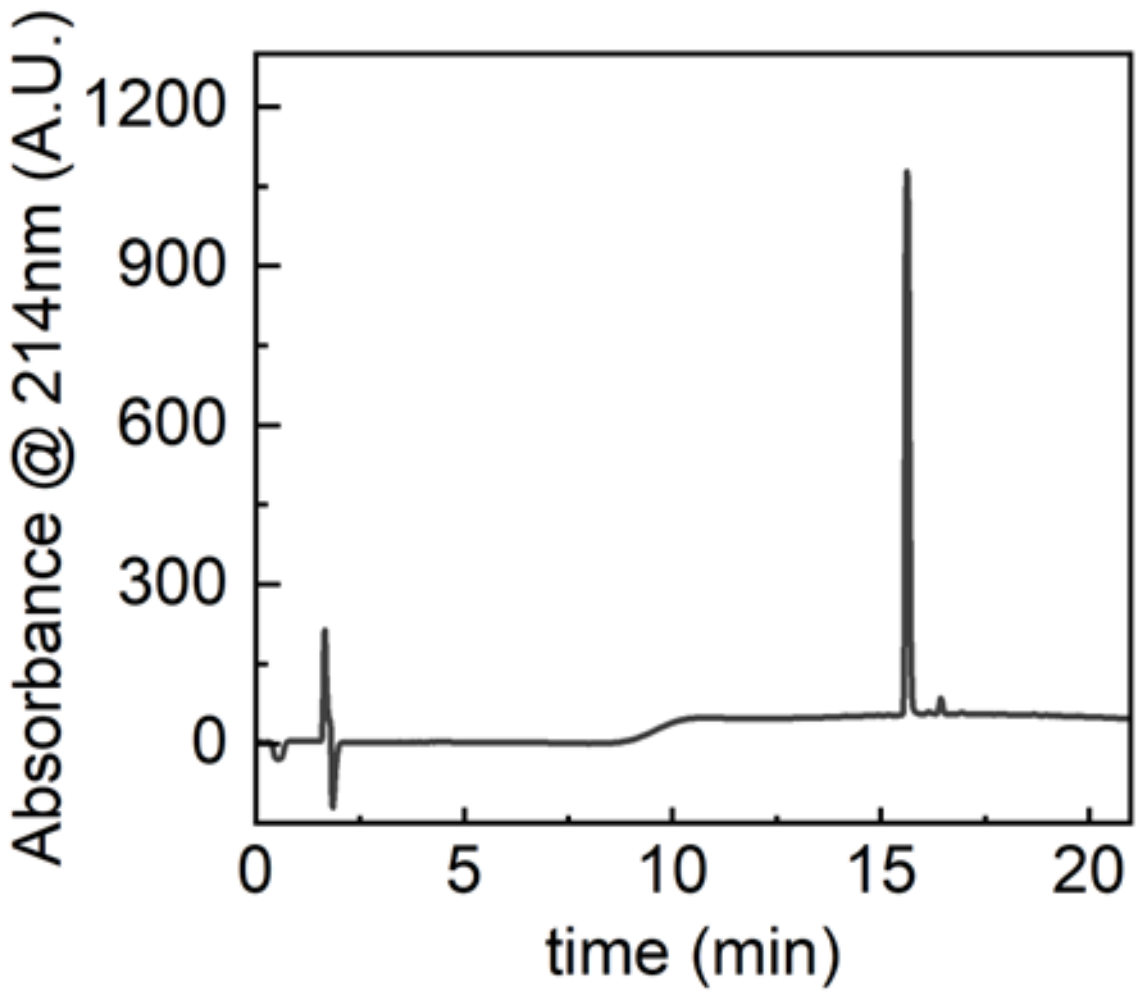

Figure S6. Analytical LC trace for lFF tripeptide. Retention time (R_t): 15.6 min.

S3. Spectroscopic and analytical LC data for 5-FU-SS-lFF conjugate (NMR, ESI-MS and LC)

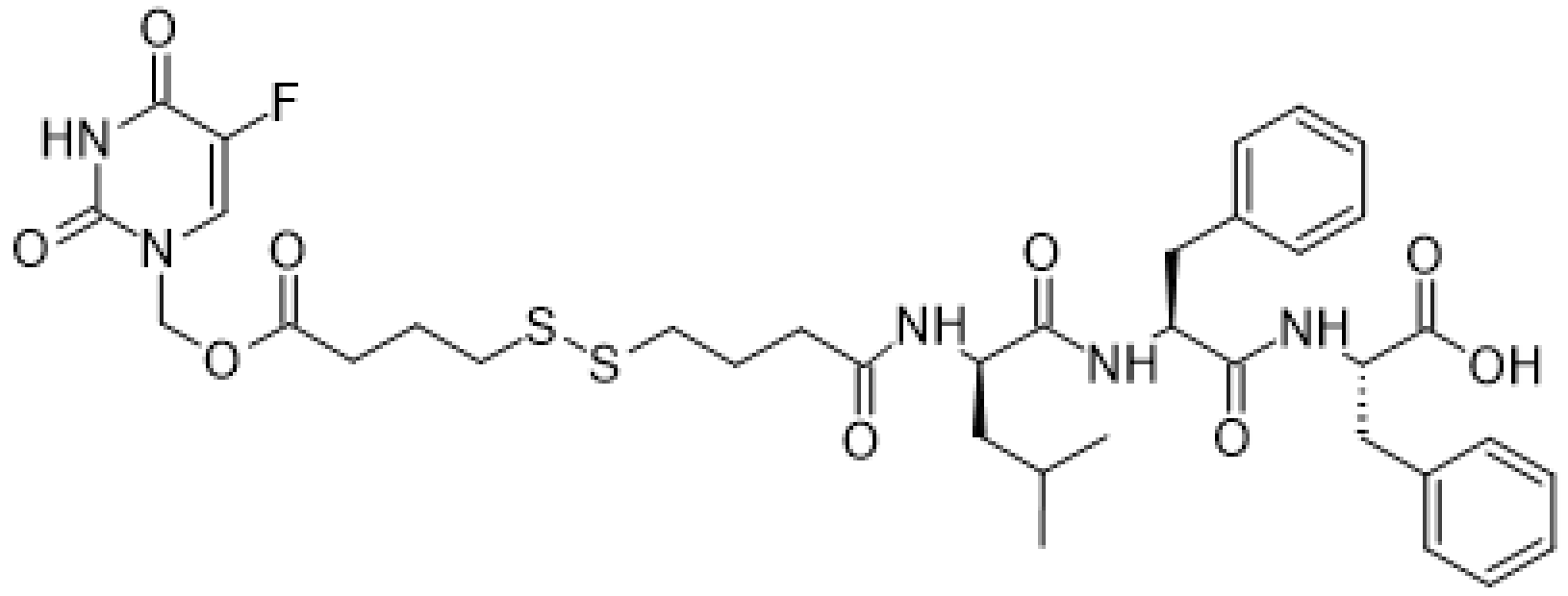

^{1}H-NMR (400 MHz, DMSO-*d_6*): δ (ppm) 12.69 (s, 1H, COOH), 11.97 (s, 1H, NH, 5FU), 8.22 (t, 2H, 2NH), 8.12 (d, *J* = 6.6 Hz, 1H, Ar, 5FU), 7.92 (d, *J* = 7.8 Hz, 1H, NH), 7.30 – 7.12 (m, 10H, Ar, Phe), 5.56 (s, 2H, ester), 4.50 (m, 1H, *α*CH), 4.40 (m, 1H, *α*CH), 4.19 (m, 1H, *α*CH), 3.13 – 3.01 (m, 2H, *β*CH_2), 2.96 (m, 1H, *β*CH_2), 2.71 – 2.61 (m, 5H, 1H *β*CH_2 and 4H CH_2 DTBA), 2.43 (d, *J* = 7.3 Hz, 2H, CH_2 DTBA), 2.18 (h, *J* = 7.4 Hz, 2H, CH_2 DTBA), 1.93 – 1.71 (m, 4H, CH_2 DTBA), 1.22 – 1.08 (m, 3H, *γ*CH and *β*CH_2), 0.71 (dd, *J* = 12.9, 6.3 Hz, 6H, *δ*CH_3); **^{13}C-NMR** (100 MHz, DMSO-*d_6*): δ (ppm) 173.6, 173.0, 172.8, 172.4, 172.1 (5 x CO), 158.5, 150.2 (2 x CO, 5FU), 141.5, 139.2, 138.8, 138.5, 130.6, 130.2, 130.2, 130.0, 129.1, 128.8, 127.3, 127.0 (Ar, Phe, 5FU), 71.5 (αC ester), 54.7, 54.6, 52.1 (3 x αC), 41.7, 38.2, 38.1 (3 x βC), 37.6, 37.4, 34.4, 32.6, 25.6, 24.8 (DTBA), 24.6, 23.6, 22.8 (1 x γC, 2 x δC). **ESI-MS** m/z 788.2 $[M+H]^+$ $C_{37}H_{46}FN_5O_9S_2$ requires 787.3.

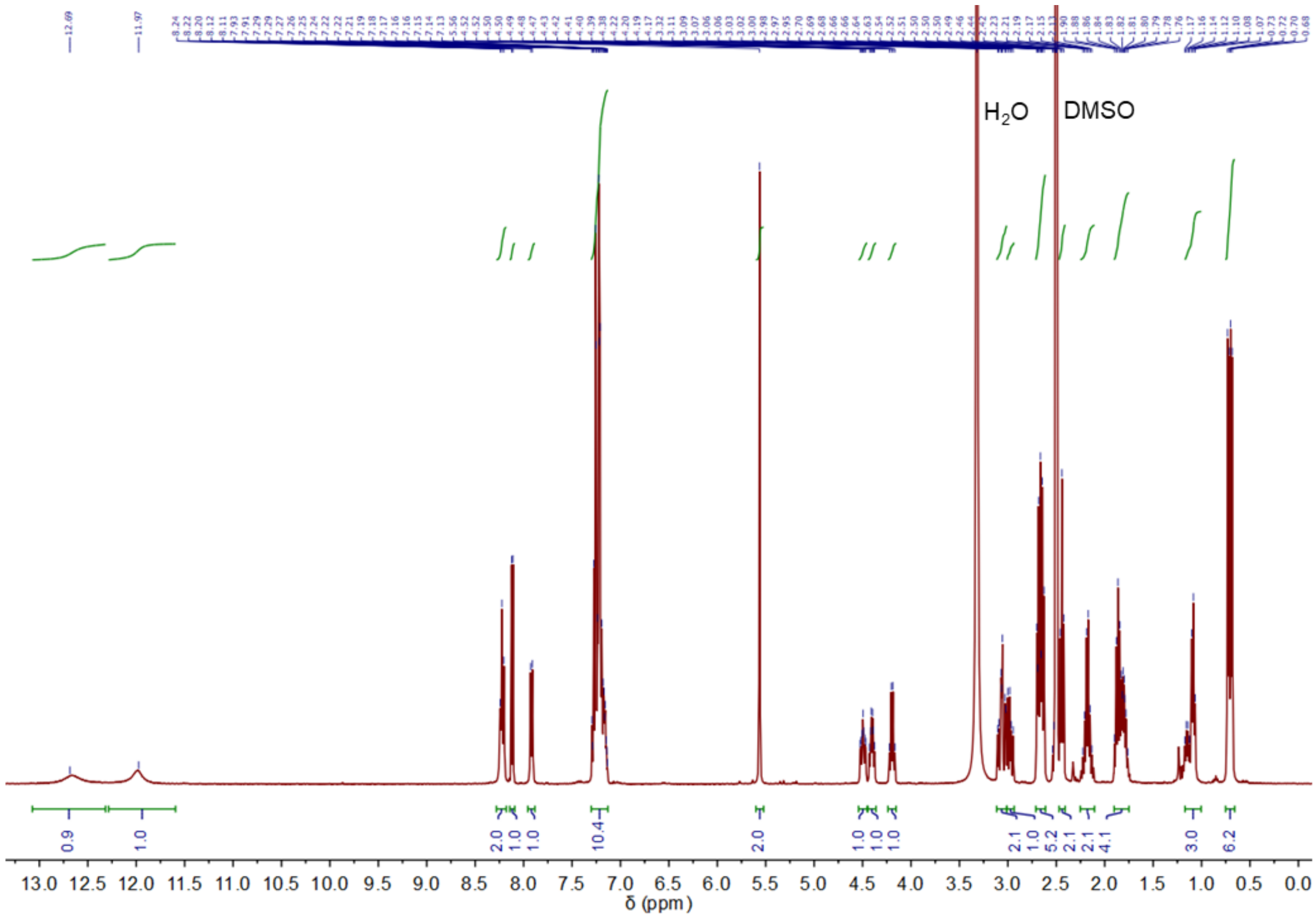

Figure S7. ^{1}H-NMR spectrum of 5-FU-SS-lFF conjugate.

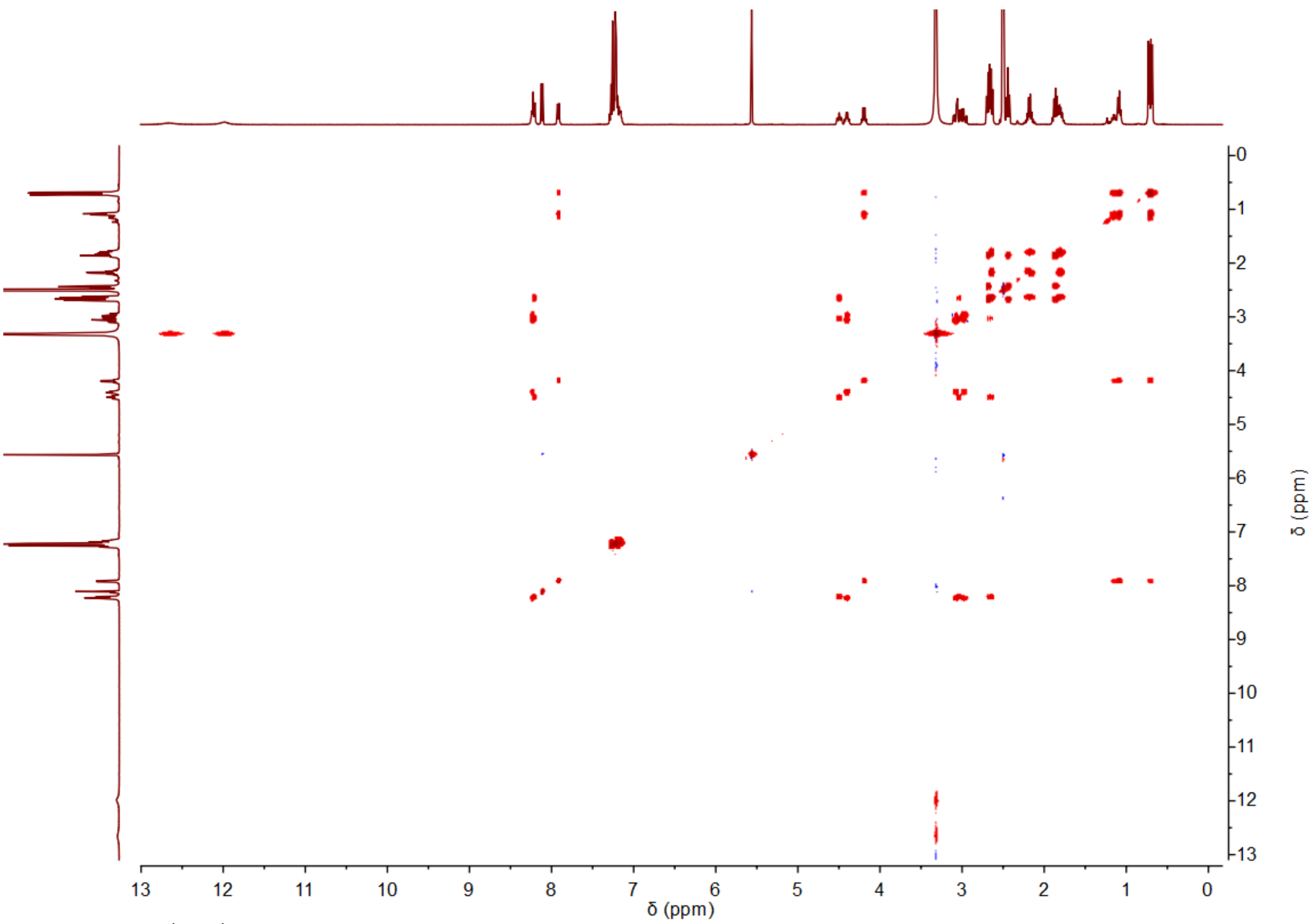

Figure S8. ^{1}H-^{1}H TOCSY 2D-NMR spectrum of 5-FU-SS-lFF conjugate.

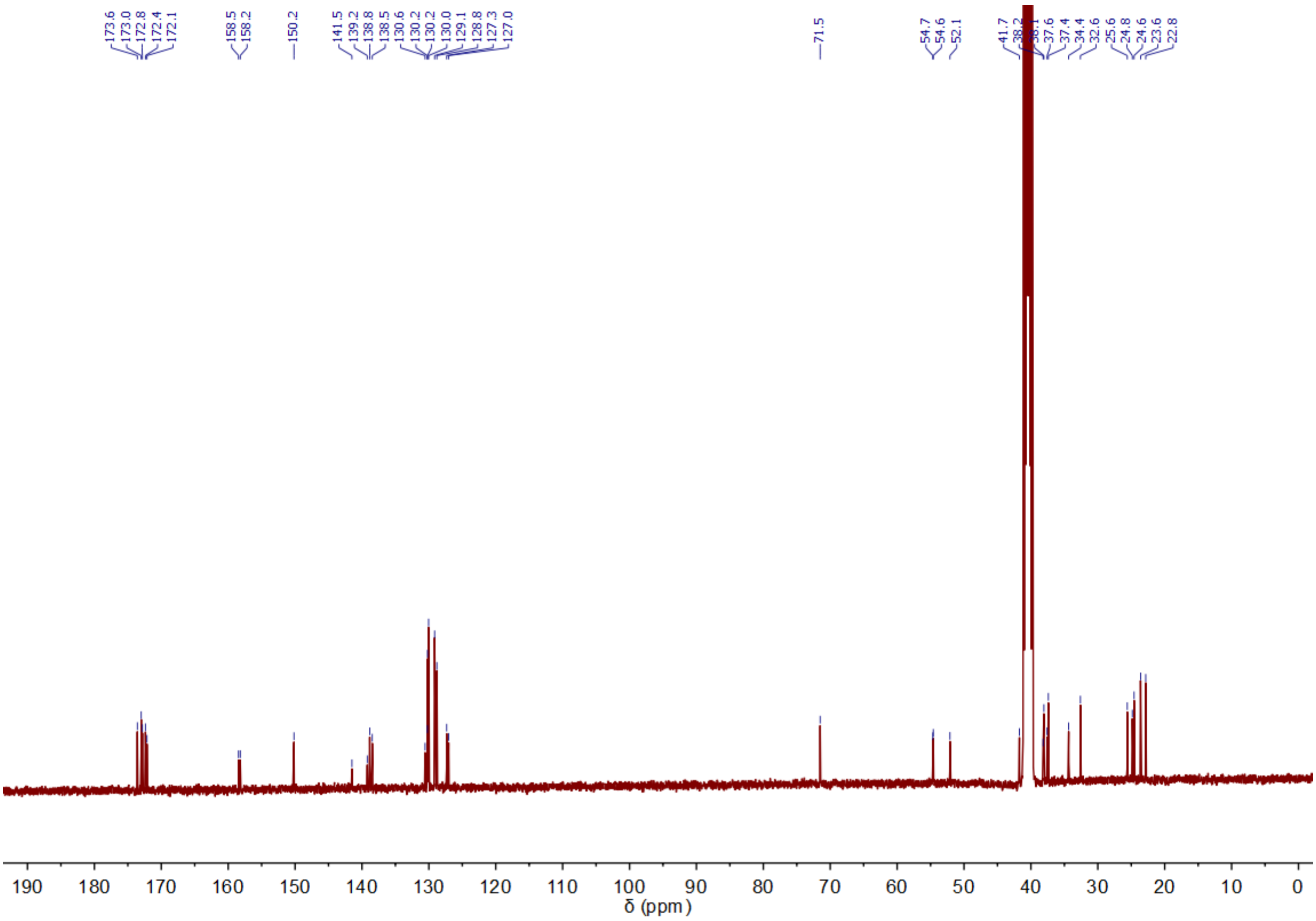

Figure S9. ^{13}C-NMR spectrum of 5-FU-SS-lFF conjugate.

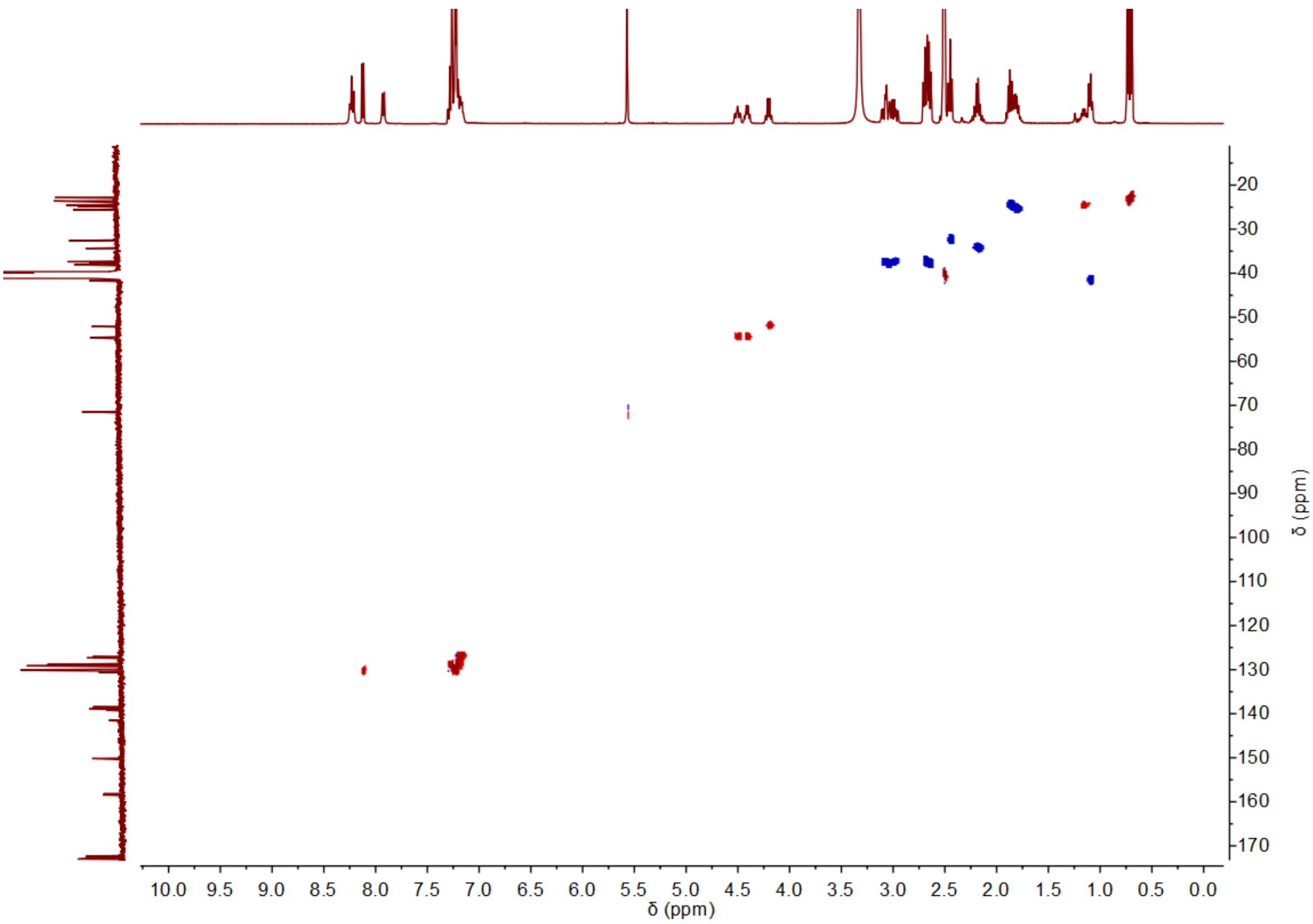

Figure S10. ^{1}H-^{13}C HSQC 2D-NMR spectrum of 5-FU-SS-lFF conjugate.

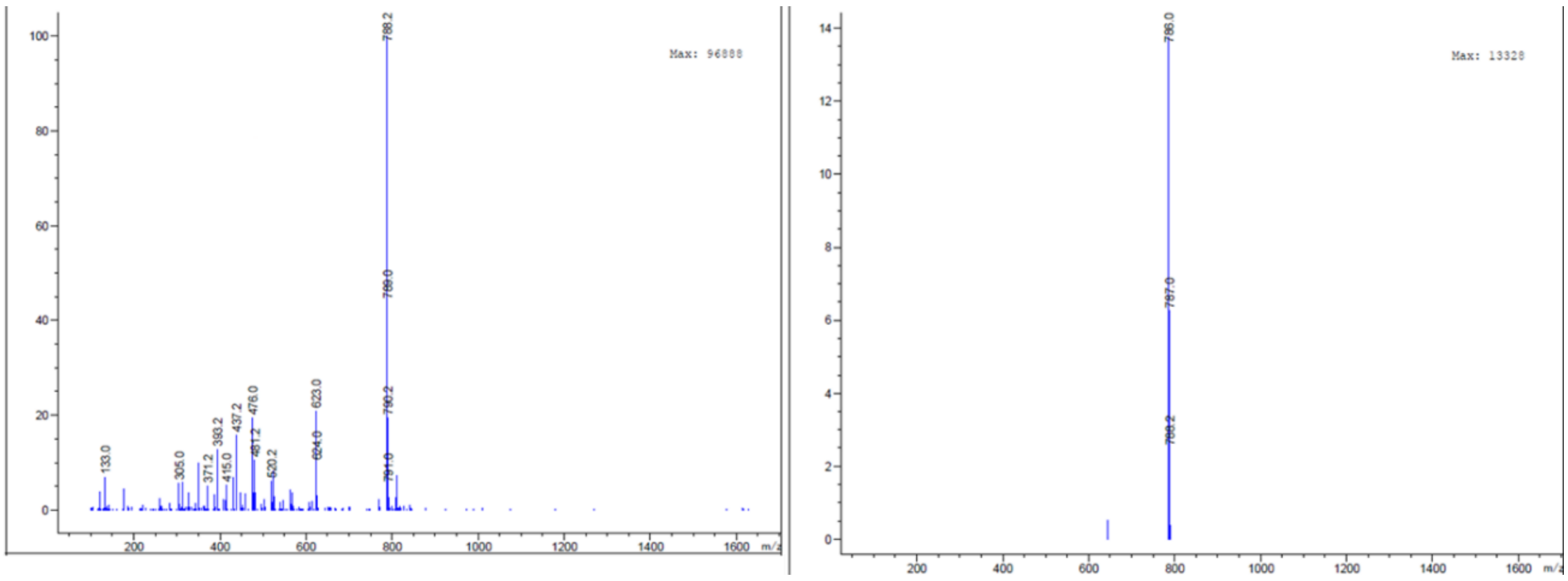

Figure S11. ESI-MS spectrum (positive and negative ion modes) for 5-FU-SS-lFF.

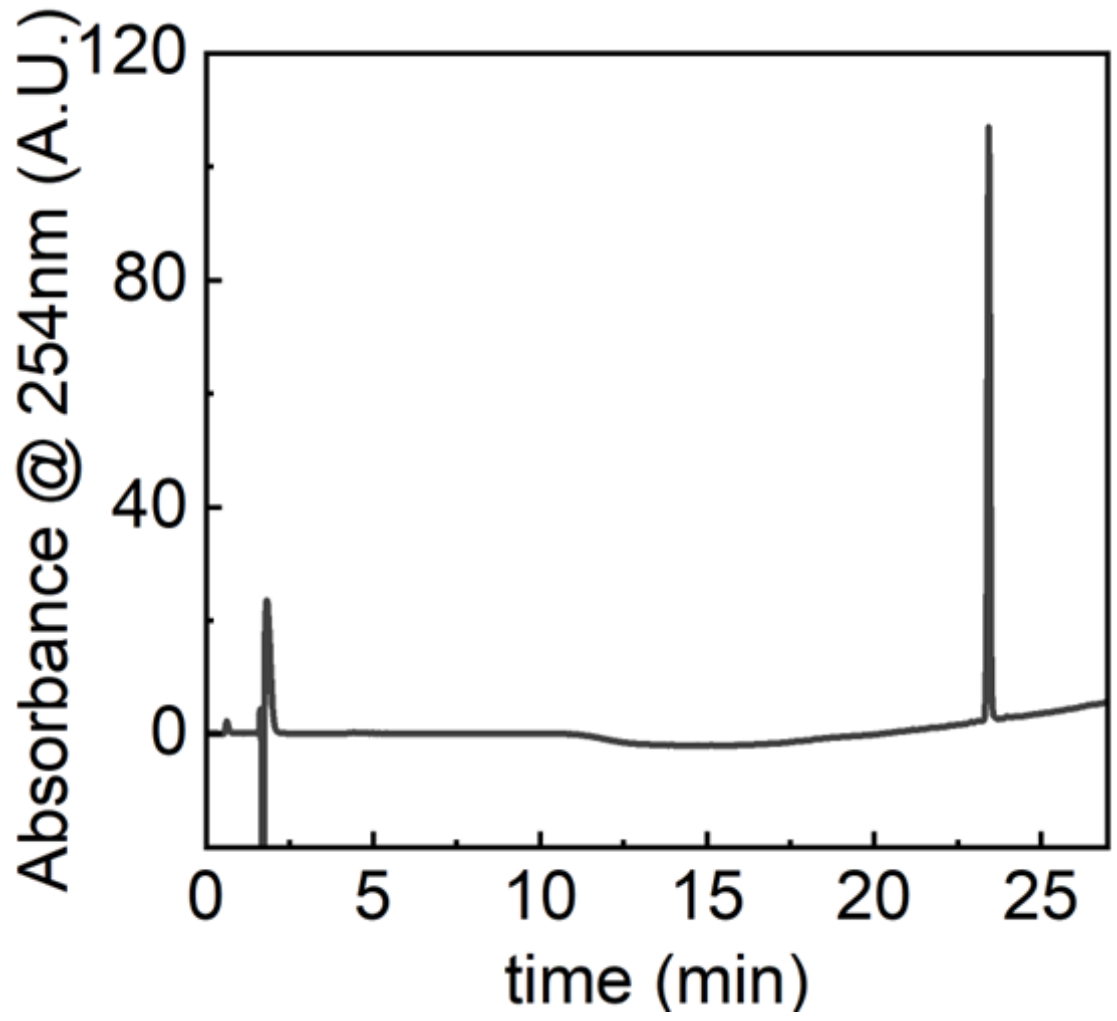

Figure S12. Analytical LC trace for 5-FU-SS-lFF conjugate. Retention time (R_t): 23.4 min.

S4. LC-MS data for drug release studies

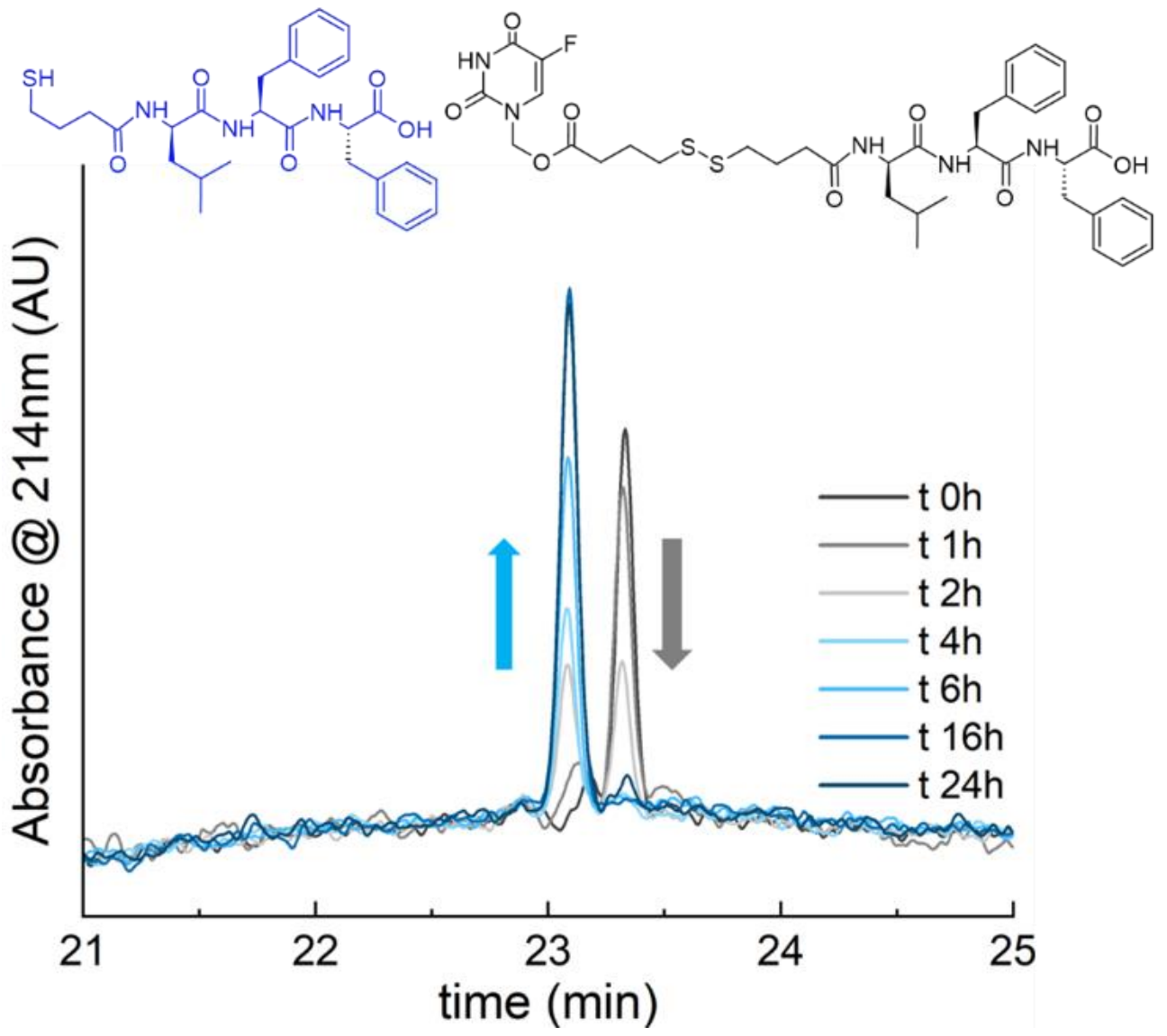

Figure S13. Overlayed LC chromatograms of 5-FU-SS-lFF hydrogel incubated in PBS (GSH 5 mM, pH 7.4). Hydrogels were disassembled at different timepoints, and the solutions were analyzed by LC-MS. Signals were recorded at 214 nm. The two peaks represent species generated from disulfide bond cleavage SH-lFF (blue), and the starting 5-FU-SS-lFF molecule (dark grey). Chromatograms were used to calculate AUC ratios relative to the highest AUC recorded (timepoint 24 h for SH-lFF and timepoint 0 h for 5-FU-SS-lFF). Note how the blue trace increases over time as opposed to the grey signal that decreases in time. Identities of the molecular structures relative to these chromatograms are reported in Figures S15 and S16. Retention times are 23.1 min for SH-lFF and 23.3 min for 5-FU-SS-lFF.

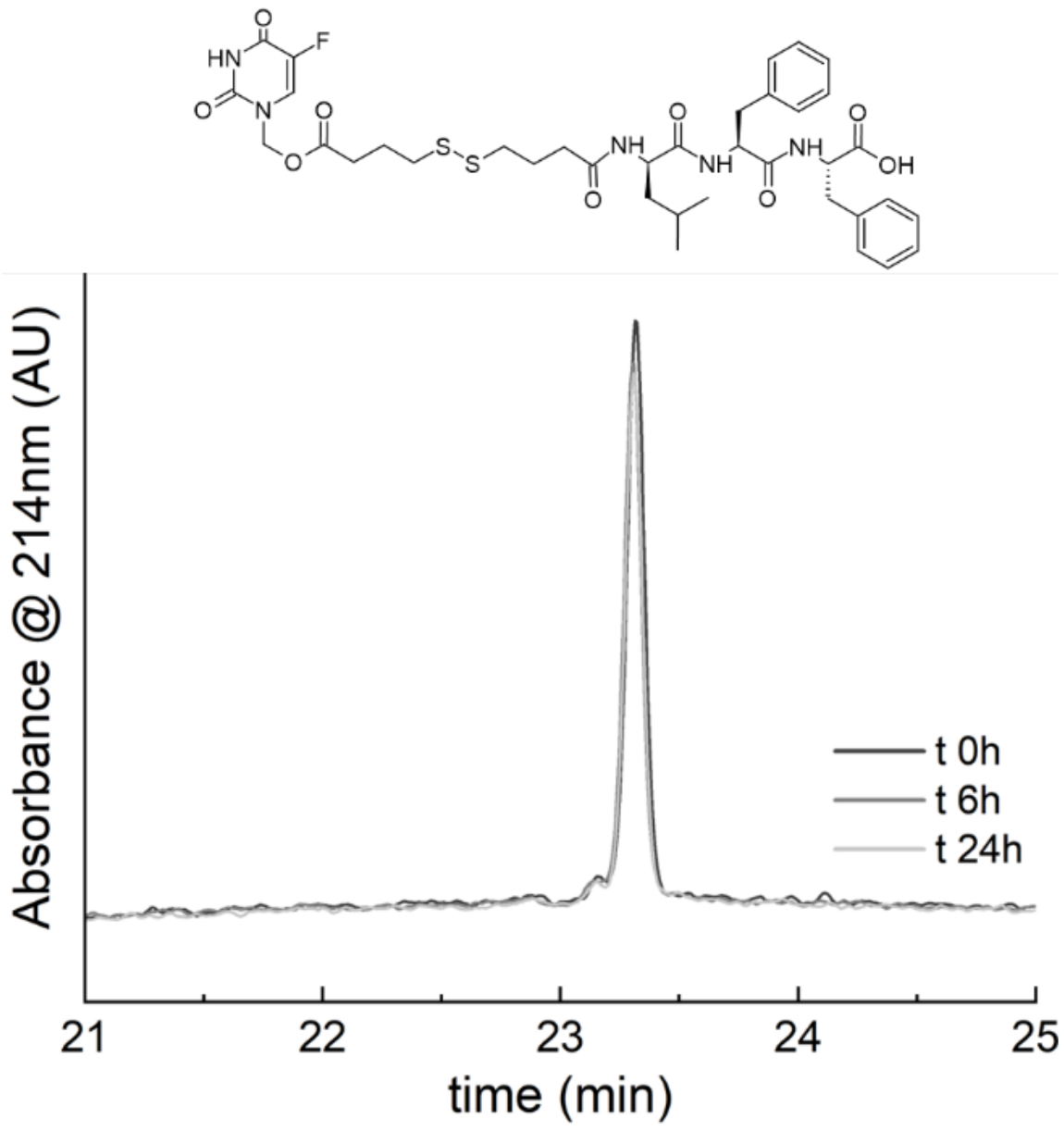

Figure S14. Overlayed LC chromatograms from 5-FU-SS-lFF hydrogel incubated in PBS, in the absence of GSH. Samples were disassembled at different timepoints, and the solutions were analysed by LC-MS (chromatograms were recorded at 214 nm). Chromatograms were used to calculate AUC ratios, relative to the highest AUC recorded at timepoint 0 h. Note how the signal relative to 5-FU-SS-lFF is almost constant in time (retention time 23.3 min). Note the absence of the peak at 23.1 min relative to SH-lFF deriving from disulfide bond cleavage.

Relative MS spectrum of the recorded species from these chromatograms is reported in Figure S16.

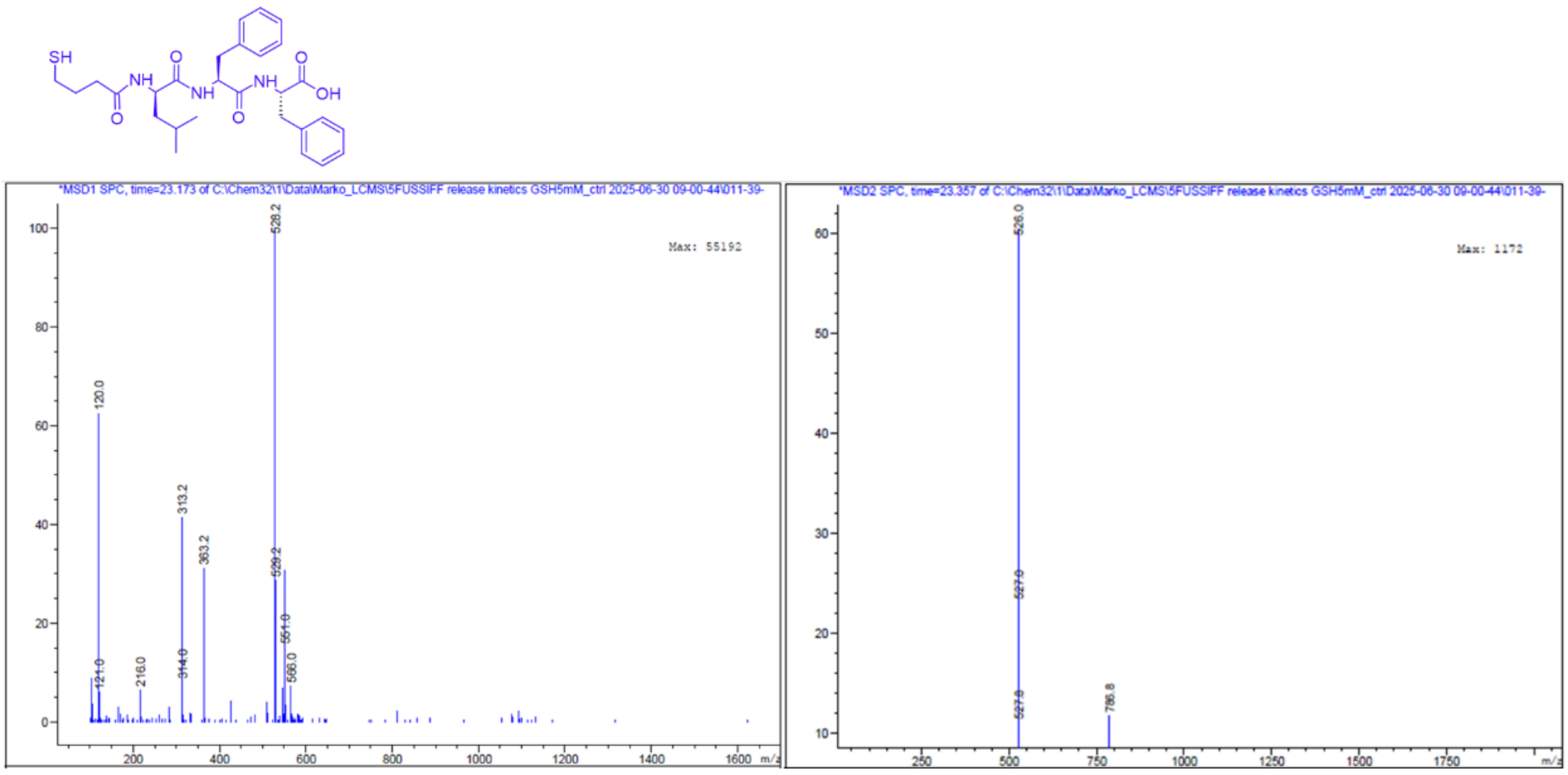

Figure S15. ESI-MS spectrum (positive and negative ion modes) for SH-lFF, (positive ion mode: m/z 528.2 $[M+H]^+$ $C_{28}H_{37}N_3O_5S$ requires 527.2).

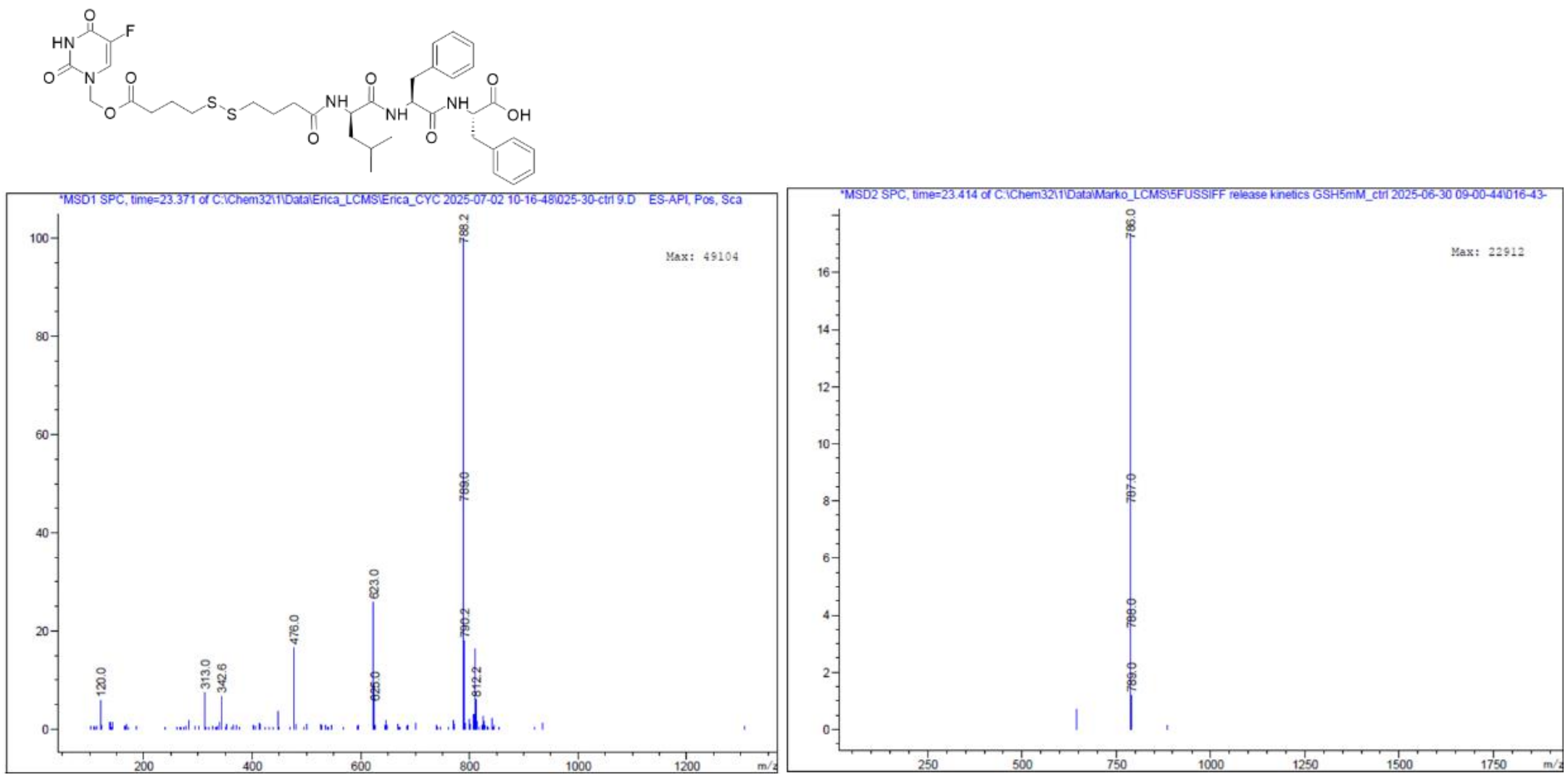

Figure S16. ESI-MS spectrum (positive and negative ion modes) for 5-FU-SS-lFF, (positive ion mode: m/z 788.2 $[M+H]^+$ $C_{37}H_{46}FN_5O_9S_2$ requires 787.3).

S5. ^{19}F-NMR of 5-FU-SS-lFF before and after removal of TFA counterion

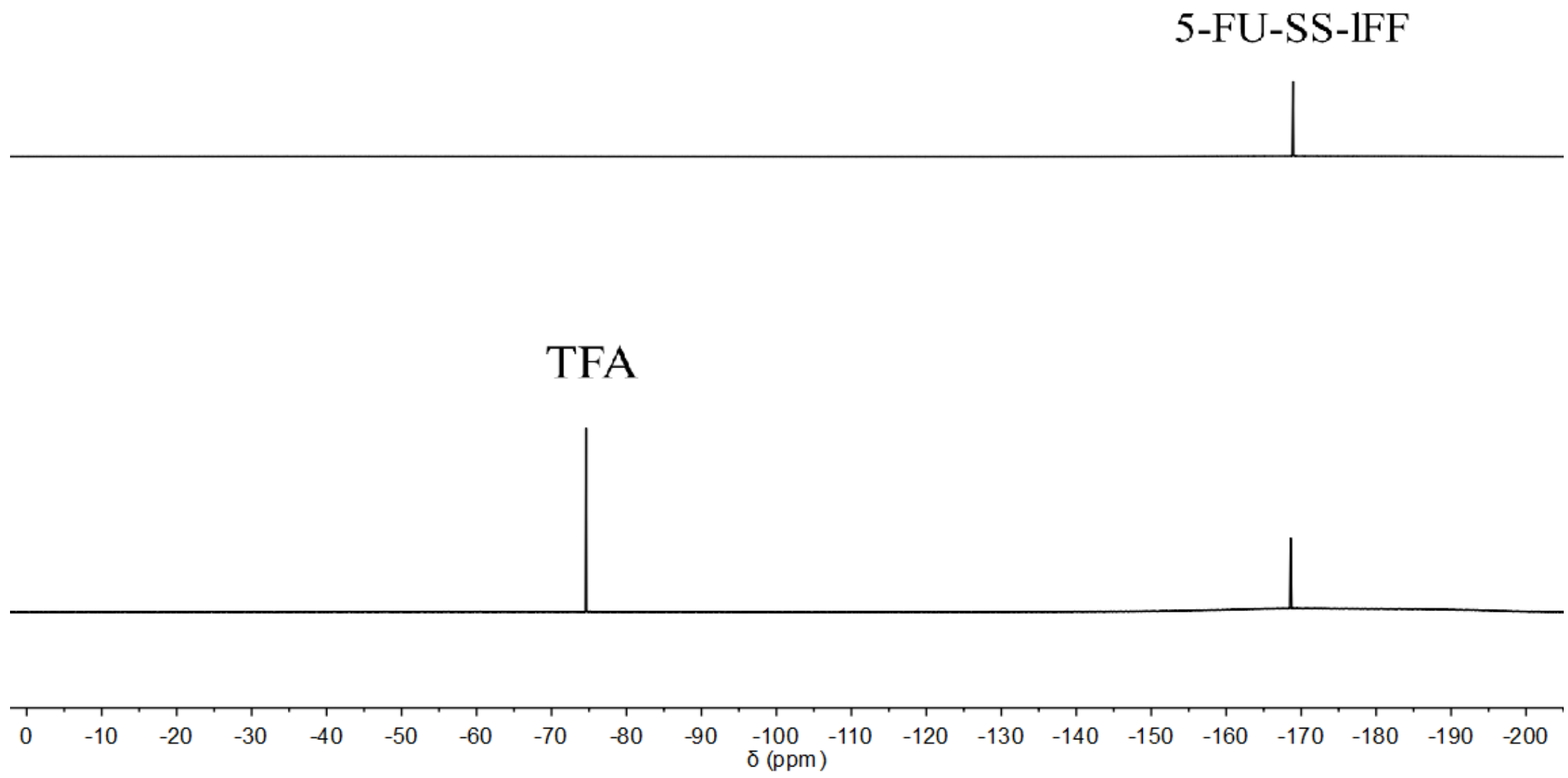

Figure S17. ^{19}F-NMR spectrum of 5-FU-SS-lFF conjugate before (bottom) and after (top) removal of TFA. Note the disappearance of the TFA signal after the sample was treated with HCl, while the peak corresponding to fluorine from 5-FU-SS-lFF is still present. The same procedure was performed for lFF tripeptide (data not shown).

S6. Cytotoxicity test using a colorectal cancer spheroid model

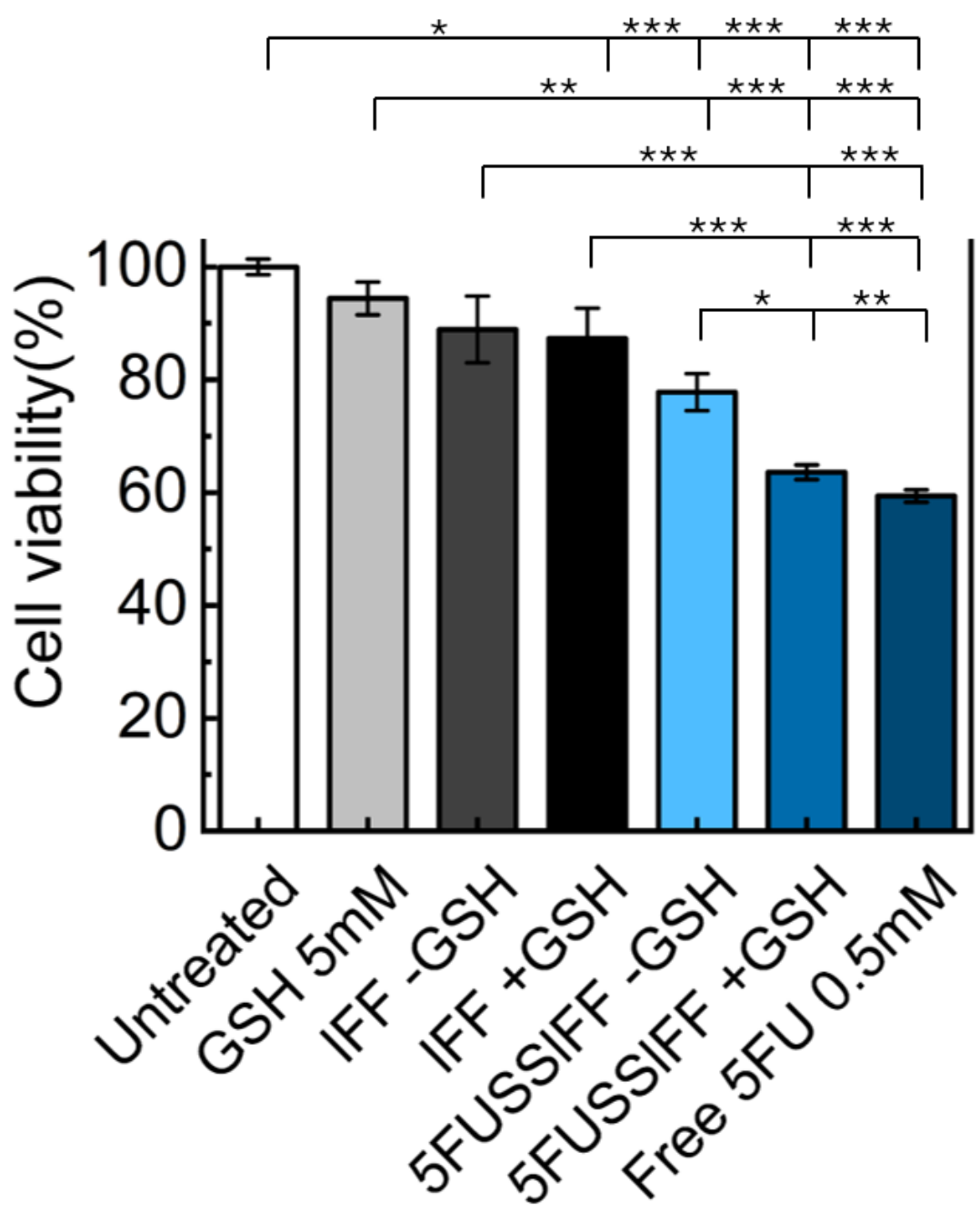

Figure S18. Anticancer effect of hydrogel formulations. The anticancer effect of lFF and 5-FU-SS-lFF hydrogels under physiological and reducing conditions (in the presence of 5 mM GSH) in human colorectal cancer spheroids (Caco-2 cell line), assessed by ATP quantification and determination of cell viability, following 24 h exposure to conditioned media from hydrogel preparations. Untreated spheroids were used as a control, and spheroids treated with 5 mM GSH were used to assess the effect of GSH alone. Free 5-FU at 0.5 mM was used as a positive control. Data are derived from three independent experiments performed in triplicate and expressed as mean±SEM. *p<0.05, **p<0.01, ***p<0.001 (1-way ANOVA followed by Dunnett's multiple comparison test, using untreated cells as a control).

S7. Cytotoxicity test using a non-tumoral cell spheroid model

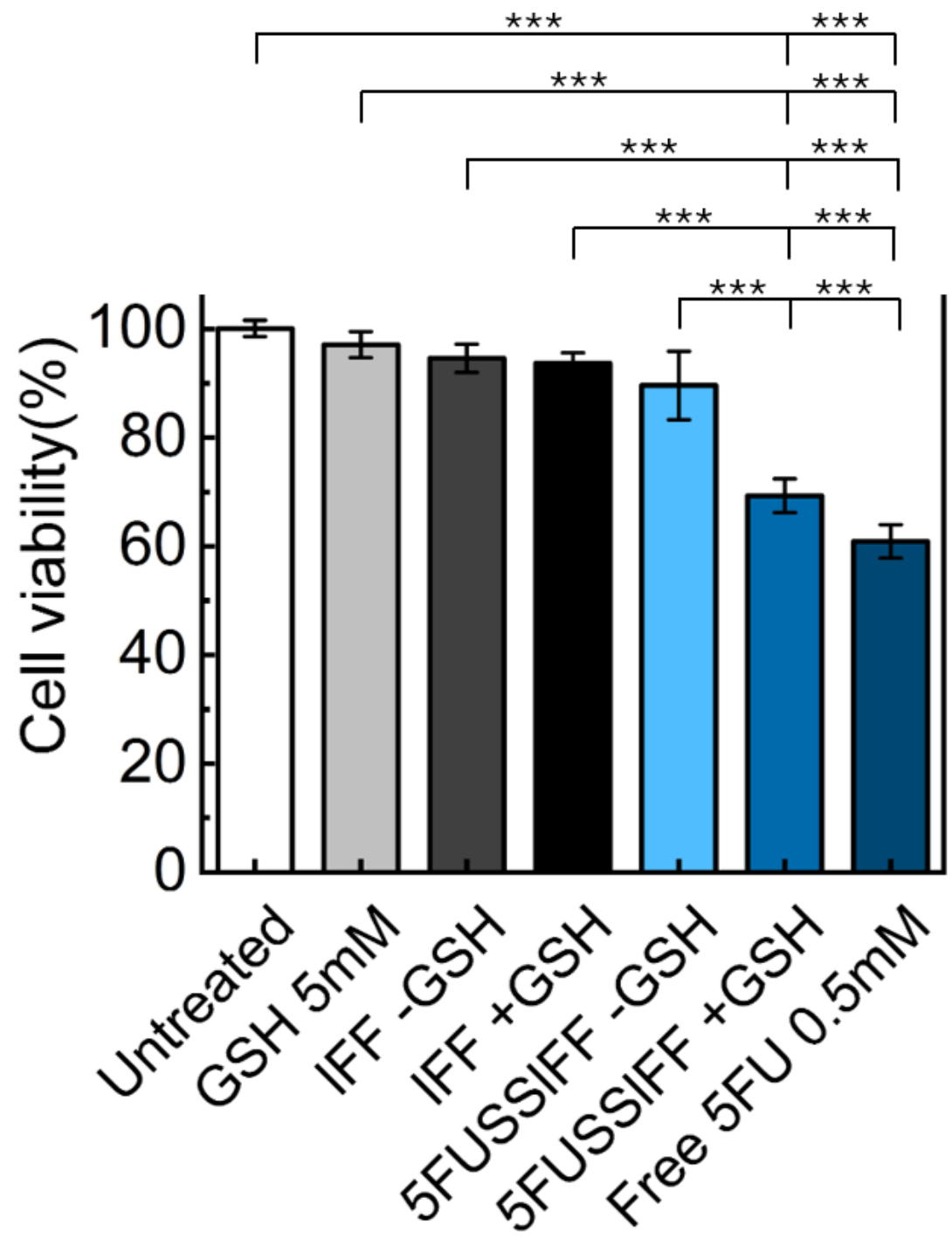

Figure S19. Cytotoxicity of hydrogel formulations on healthy control. The cytotoxicity of lFF and 5-FU-SS-lFF hydrogels under physiological and reducing conditions (in the presence of 5 mM GSH) in MDCK II non-tumoral spheroids (MDCK II non-tumoral epithelial cell line), assessed by ATP quantification and determination of cell viability, following 24 h exposure to conditioned media from hydrogel preparations. Untreated spheroids were used as a control, and spheroids treated with 5 mM GSH were used to assess the effect of GSH alone. Free 5-FU at 0.5 mM was used as a positive control. Data are derived from three independent experiments performed in triplicate and expressed as mean±SEM. ***$p<0.001$ (1-way ANOVA followed by Dunnett's multiple comparison test, using untreated cells as a control).
